# Circadian ontogenetic metabolomics atlas: an interactive resource with insights from rat plasma, tissues, and feces

**DOI:** 10.1007/s00018-025-05783-w

**Published:** 2025-06-28

**Authors:** Lucie Rudl Kulhava, Pavel Houdek, Michaela Novakova, Jiri Hricko, Michaela Paucova, Ondrej Kuda, Martin Sladek, Oliver Fiehn, Alena Sumova, Tomas Cajka

**Affiliations:** 1 Institute of Physiology of the Czech Academy of Sciences, Videnska 1083, Prague, 14200 Czech Republic; 2 University of California, Davis, 451 Health Sciences Drive, Davis, CA 95616 USA

**Keywords:** Metabolomics, Lipidomics, Circadian rhythm, Atlas, Resource

## Abstract

Circadian rhythms regulate key physiological processes through clock genes in central and peripheral tissues. While circadian gene expression during development has been well studied, the temporal dynamics of metabolism across tissues remain less understood. Here, we present the Circadian Ontogenetic Metabolomics Atlas (COMA), which maps circadian metabolic rhythms across 16 rat anatomical structures. The brain (suprachiasmatic nuclei, medial prefrontal cortex) and periphery (liver, plasma) span developmental stages from embryonic E19 to postnatal P2, P10, P20, and P28. Fecal samples include all four postnatal stages, while additional peripheral tissues were analyzed at P20 and P28. Using a multiplatform liquid chromatography–mass spectrometry approach, we annotated 851 metabolites from 1610 samples. We identified distinct circadian shifts, particularly during the transition from nursing to solid food intake (P10–P20), with an average of 24% of metabolites exhibiting circadian oscillations across sample types, as determined by JTK_CYCLE. Our study also underscores the importance of standardized sampling, as metabolite intensities fluctuate with both circadian rhythms and development. COMA serves as an open-access resource (https://coma.metabolomics.fgu.cas.cz) for exploring circadian metabolic regulation and its role in developmental biology.

**Graphical abstract:**

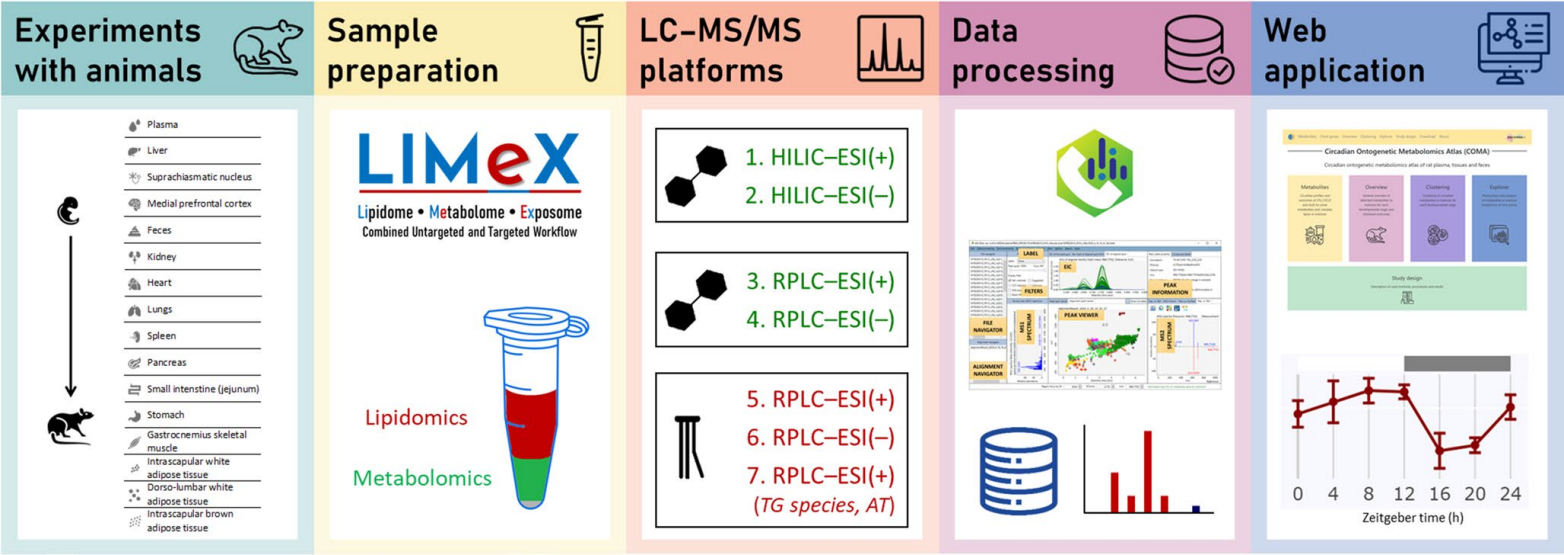

**Supplementary Information:**

The online version contains supplementary material available at 10.1007/s00018-025-05783-w.

## Introduction

The circadian rhythm, an approximately 24-hour physiological cycle, is ubiquitous across many organisms [1–3]. In mammals, it is orchestrated by a complex system involving a central clock located in the suprachiasmatic nuclei (SCN) of the hypothalamus, along with peripheral clocks distributed across neural structures and within peripheral tissues and organs [4–6]. The development of these circadian clocks during the prenatal period depends on the intricate differentiation and maturation of specific anatomical structures [7]. For example, in rats, neurogenesis within the SCN begins around embryonic day (E)14 and continues until E17 [8, 9]. This neurogenic process originates from a specialized zone within the ventral diencephalic germinal epithelium, which is part of the periventricular cell group. Although neurogenesis is complete by E18, morphological maturation of SCN neurons continues progressively until postnatal day (P)10 [10, 11].

In adults, the circadian system is hierarchically organized, with the central clock synchronizing subordinate clocks throughout the body, which is crucial for adapting physiological functions to cyclic external conditions. This system undergoes substantial developmental changes, including shifts in its responsiveness to external cues [12]. During the prenatal period, maternal signals primarily entrain the developing clock, whereas postnatally, the light–dark cycle becomes the dominant zeitgeber. The central clock acquires its functional properties gradually through a programmed process [13, 14], eventually entraining peripheral clocks that exhibit autonomous rhythmicity at different developmental stages [15]. In early development, maternal feeding is a primary driver of peripheral clock entrainment. As development progresses, the central clock becomes fully functional and takes over this regulatory role [11].

Despite extensive research primarily focused on gene expression, the development of circadian rhythmicity remains a subject of ongoing exploration [16]. Recent findings suggest cellular coupling and tissue-wide synchronization of single-cell rhythms may not occur until late in embryogenesis [10]. Our research and others have provided evidence that, throughout development, the phase of clock gene expression rhythms in certain tissues gradually shifts until reaching the adult stage [17, 18]. However, despite these advances, studies on circadian rhythmicity at the metabolite level during development remain scarce. Although transcriptomics has illuminated a substantial portion of the genome regulated by the molecular clock, metabolomics has been limited by the lack of robust analytical platforms and bioinformatics tools

Nevertheless, in the past decade, it has been shown that chemical profiling of biological specimens using mass spectrometry-based metabolomics is valuable in revealing the influence of the circadian clock on both mouse and human metabolism [19–22]. The circadian metabolome has been mapped under various conditions, such as sleep deprivation [23–25], jet lag [26, 27], exercise [28, 29], genetic perturbation [30, 31], different states of neuronal excitability [32], aging [33–35], acute or chronic cold exposure [36, 37], and nutritional challenges [38–40]. Most of these studies have analyzed plasma or serum, given their accessibility and role as a critical link between peripheral tissues. Recently, mapping metabolite dynamics over 24 h in plasma and multiple tissues, including liver, muscle, medial prefrontal cortex (mPFC), SCN, sperm, white adipose tissue (WAT), and brown adipose tissue (BAT) in response to chow and high-fat diets has been reported to provide important temporal insights [38]. Similarly, another study profiled serum and several tissues (muscle, liver, hypothalamus, heart, WAT, and BAT) following acute exercise performed at different times of the day [28].

Compared to circadian gene expression profiles, comprehensive bioinformatics resources mapping the circadian metabolite profiles in humans, mice, and rats are rare. The CircadiOmics portal, originally developed for transcriptomic data, was recently expanded to include metabolomic data from various mouse tissues analyzed using a multiplatform liquid chromatography–mass spectrometry (LC–MS) approach [38]. This resource allows users to explore hundreds of metabolites with circadian regulation, facilitating hypothesis generation and validation. A few datasets are available for download as supplementary materials from published studies [38, 41]. While not explicitly focused on the circadian metabolome, some attempts have been made to create metabolome databases for multiple tissues [42, 43]. However, these studies relied on a single analytical platform, capturing only a subset of the metabolome. This underscores the insufficient data on the circadian metabolome characterizing diverse tissues, emphasizing the need for easily accessible and reusable resources for future studies, as we recently highlighted in our discussion of metabolomics atlases [44].

To address this gap, we aimed to create an interactive, open-access atlas focused on the ontogenetic development of rats. This resource provides comprehensive metabolomics data across plasma, multiple tissues, and feces using a multiplatform LC–MS approach. Furthermore, by sharing raw LC–MS instrumental files, we facilitate retrospective data mining, potentially leading to additional annotations for unknown metabolites through spectral library search or the discovery of novel metabolites.

## Materials and methods

### Experiments with animals

Three-month-old male and female Wistar: Han rats (Institute of Physiology of the Czech Academy of Sciences) were kept under a 12:12 h light–dark cycle (lights on at 06:00 a.m., designated Zeitgeber time 0) at 21 ± 2 °C with free access to food and water. Overhead 40 W fluorescent tubes provided light, resulting in illumination levels of 150 lx, varying based on cage position in the animal room. Female rats were mated with males, and those with positive sperm in vaginal smears were individually housed.

Fetuses were collected from the first group at embryonic day E19, while other groups were studied for postnatal development. After delivery on postnatal day 0 (P0), dams and their pups were maintained under a 12:12 h light–dark cycle, with the pups undisturbed and raised by their mother during lactation (P0–P20) on a standard diet (pellets). To collect the samples during the 24-hour cycle, fetuses were killed by rapid decapitation, and pups were euthanized at P2, P10, P20, and P28 with an overdose of thiopental (50 mg/kg, i.p.). Tissues (SCN, mPFC, liver) and plasma were collected for all five developmental stages. Feces were collected for P2, P10, P20, and P28 stages. For the P20 and P28 stages, additional tissues (kidney, heart, lungs, spleen, pancreas, small intestine (jejunum), stomach, gastrocnemius skeletal muscle, intrascapular WAT (isWAT), dorso-lumbar WAT (dlWAT), and intrascapular BAT (iBAT)) were collected. All samples were collected at Zeitgeber times 0, 4, 8, 12, 16, 20, and 24 h, each with five replicates. Samples for LC–MS were promptly stored at − 80 °C until further processing and analysis, with plasma prepared from abdominal/thoracic blood using EDTA collection tubes. For the reverse transcription-quantitative polymerase chain reaction (RT qPCR) method, dlWAT samples were immersed in RNAlater (Merck), pancreas samples were immediately homogenized using ceramic beads in RNA isolation buffer (GenElute Total RNA kit, Merck), and whole brains were frozen in dry ice before storage at − 80 °C. Brains were sectioned on cryocut, and SCN samples were dissected using laser-capture microdissection as described previously [45].

### LC–MS-based metabolomics

For the sample extraction, a biphasic solvent system of methanol, methyl *tert*-butyl ether, and water was used to isolate complex lipids and polar metabolites. Six different LC–MS platforms [46] were used for profiling plasma, non-fat tissues, and feces with optimized conditions described before [47]: (1) hydrophilic interaction chromatography (HILIC) metabolomics in positive electrospray ionization mode (ESI(+)), (2) HILIC metabolomics in negative electrospray ionization mode (ESI(−)), (3) reversed-phase liquid chromatography (RPLC) metabolomics in ESI(+), (4) RPLC metabolomics in ESI(−), (5) RPLC lipidomics in ESI(+), and (6) RPLC lipidomics ESI(−), and (7) additional RPLC lipidomics analysis in ESI(+) on adipose tissues to detect abundant triacylglycerols.

Details about the sample extraction methods for each matrix, LC–MS analysis conditions, enhanced MS/MS spectra acquisition for metabolite annotation, quality control procedures, and LC–MS data processing are provided in the Supplemental Information. A list of annotated metabolites can be found in Table S1.

### RT qPCR

The RT qPCR method was used to detect *Per1*, *Per2*, *Nr1d1/Rev-Erbα*, *Nfil3/E4bp4*,* Dbp*,* Cry1*, and *Bmal1* mRNA levels in selected tissues (3–5 replicates × group × time point). First, RNA was isolated using GenElute Total RNA kit (Merck, peripheral tissues) or RNeasy Micro kit (Qiagen, SCN), up to 0.5 µg was then reverse-transcribed using a High Capacity cDNA RT Kit (ThermoFisher). Diluted cDNA was then amplified on LightCycler480 (Roche) using SYBR Select qPCR Master Mix (ThermoFisher) as described previously [45, 48–50]. Liver samples were analyzed previously [18] and are included in the COMA dataset for convenience.

### Statistical analysis and data visualization

JTK_CYCLE, designed to identify and characterize cycling variables in large datasets, was used [51]. The metabolomics data were log_10_ transformed and median normalized, followed by running the R script JTK_CYCLE v.3 with parameters: timepoints = 7, reps = 5, periods = 6, interval = 4. Metabolites with permutation-based *p*-values (*ADJ.P*) < 0.05 were considered statistically significant [41]. Reported *p*-values (*ADJ.P*) are, by default, Bonferroni-adjusted for multiple testing [51]. For “Outlier-free” analysis, data were log_10_ transformed, and for each group of 5 biological replicates, data points outside ±4 times the median absolute deviation were considered outliers and removed. For clustering analysis, data were log_10_ transformed and *z*-score normalized. For statistically significant metabolites (JTK_CYCLE, *p* < 0.05), the profiles were fitted to one of the six key clusters, with each graph displaying the number of metabolites in the cluster and their average Pearson correlation coefficient. Data from RT qPCR as relative expression were not further normalized.

Differential RhythmicitY analysis in R (*dryR*), developed to analyze rhythmicity in datasets comprising several conditions, was also used [52]. The metabolomics data were log_10_ transformed and median normalized, followed by execution of the R script dryR. The script (using f_24 function) was run separately for each matrix × developmental stage combination, reporting *p*-values corrected for multiple testing using the Benjamini–Hochberg method (*padj*). For the late developmental stages P20 and P28, the script was also run in parallel (using drylm function) to report rhythmicity models across these two conditions (Table S2), where model codes represent: 0 — no data available for calculation, 1 — non-rhythmic, 2 — loss of rhythm, 3 — gain of rhythm, 4 — unaltered rhythm, and 5 — altered rhythm. The distinction between models 4 and 5 is based on fitting the equation *y*(*t*) = *µ* + *α*cos(*ωt*) + *β*sin(*ωt*), with identical *α* and *β* coefficients for model 4, and differing coefficients for model 5.

For multivariate analyses such as principal component analysis (PCA) and partial least squares-discriminant analysis (PLS-DA), the metabolomics data were log_10_ transformed and Pareto-scaled. For PCA, score and loading plots are provided, while for PLS-DA, variable importance in projection (VIP) scores are also reported. PLS-DA models were further assessed by 5-fold cross-validation, reporting the *Q*² performance measure (an estimate of the model’s predictive ability) and permutation test results (*n* = 1000), yielding an empirical *p*-value.

The final dataset of polar metabolites, complex lipids, and clock genes for all samples was visualized in Plotly using a Python-based Flask web application. Circadian profiles are expressed as mean ± standard deviation for a particular group × time point.

### Website implementation

Circadian Ontogenetic Metabolomics Atlas (COMA) is an interactive web application built using the Flask framework (https://flask.palletsprojects.com). The app uses an SQLite database (https://www.sqlite.org) accessed via Flask-SQLAlchemy for efficient data storage and management. The front end is styled with Bootstrap (https://getbootstrap.com), CSS (Cascading Style Sheets), and JavaScript. For visualizations, it uses Plotly (https://plotly.com/python), providing dynamic, interactive graphs. The app supports scientific analysis with machine learning algorithms from scikit-learn (https://scikit-learn.org), including PCA and PLS-DA, and integrates the JTK_CYCLE (https://sites.wustl.edu/hugheslab/jtk_cycle) and dryR algorithms (https://github.com/naef-lab/dryR) for detecting rhythmic components in the data.

## Results

### Building circadian ontogenetic metabolomics atlas (COMA)

**Fig. 1:**
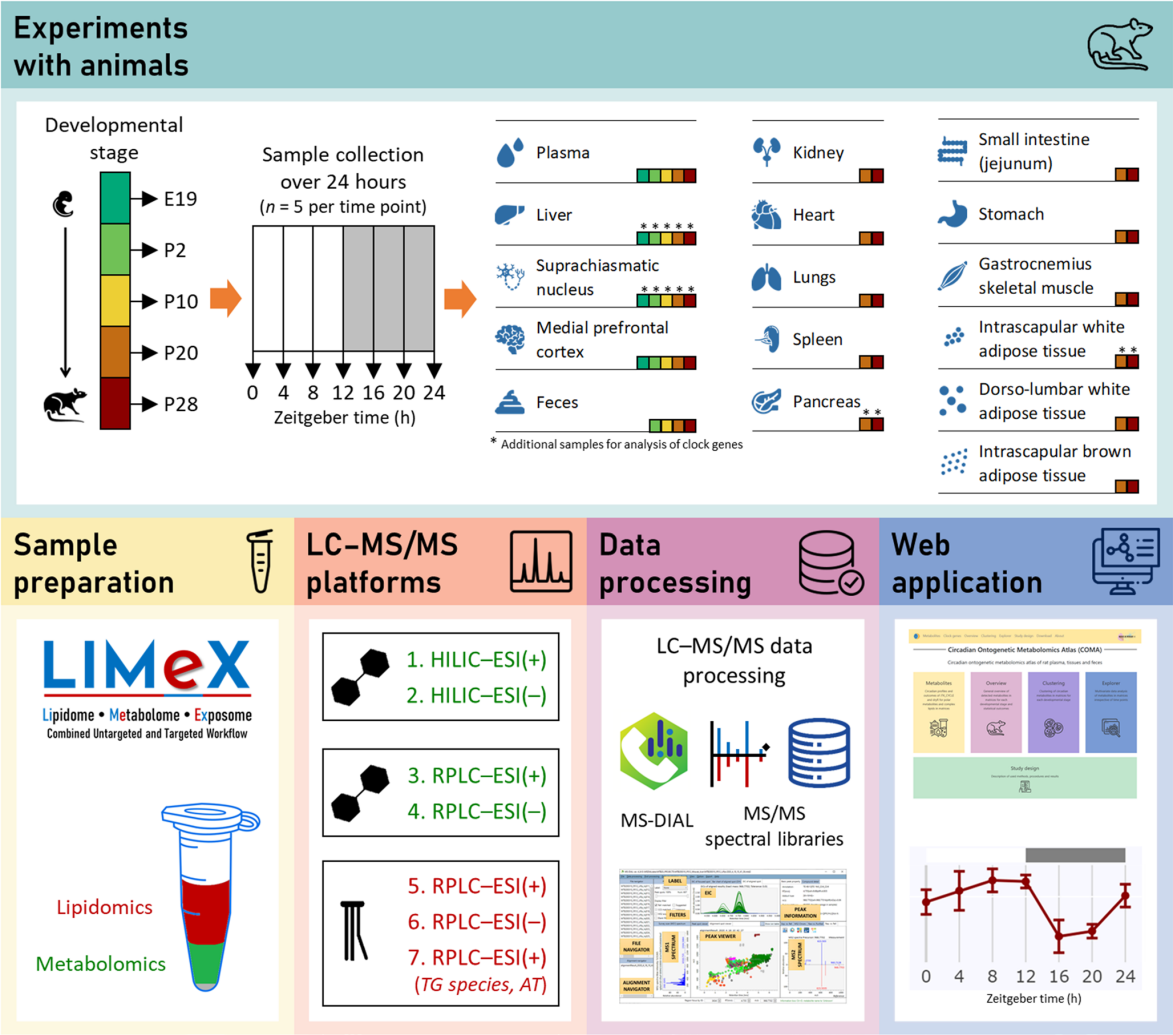
Graphic illustration of the workflow toward building circadian ontogenetic metabolomics atlas of rat plasma, tissues, and feces

We have studied the development of the circadian metabolome of rat brain, specifically in the SCN and mPFC, as well as in peripheral tissues such as the liver and plasma. This study spans five developmental stages, including embryonic E19 and postnatal stages P2, P10, P20, and P28 (see Fig. 1). Fecal samples were collected at all postnatal stages. For postnatal stages P20 and P28, additional tissues were collected, including the kidney, heart, lungs, spleen, pancreas, small intestine (jejunum), stomach, gastrocnemius skeletal muscle, isWAT, dlWAT, and iBAT. Sampling was conducted at Zeitgeber times 0, 4, 8, 12, 16, 20, and 24 h for all developmental stages. Following a well-established approach, clock gene analysis was also performed for SCN, liver, pancreas, and dlWAT.

As the construction of comprehensive mass spectrometry-based metabolomics and lipidomics atlases is an emerging field [44], we provide detailed information below and in the experimental section on sample preparation, instrumental platforms, data processing and curation, as well as quality control.

We analyzed 1610 study samples using untargeted LC–MS-based metabolomics platforms, accompanied by method blanks and quality control samples. To reduce extract complexity, we employed an “all-in-one” extraction approach (LIMeX) with methanol, methyl *tert*-butyl ether, and water [46, 47], resulting in two phases: the upper one containing nonpolar metabolites (complex lipids) and the bottom one mostly consisting of polar metabolites. The optimal plasma volume, tissue amount for extraction, collected aliquots, resuspension solvent volumes, and injection volumes were determined during a pilot study. The final injection volumes were confirmed using quality control samples before analyzing all study samples.

Each of these fractions underwent analysis under different separation conditions: HILIC for highly polar metabolites such as amino acids, biogenic amines, sugars, nucleotides, acylcarnitines, and sugar phosphates; RPLC for medium polar metabolites; and RPLC analysis of complex lipids. The optimal conditions of each LC–MS platform were based on our previously published methods [47], incorporating a high-throughput approach with each sample analyzed in less than 5 min [46].

LC–MS/MS raw data were processed using MS-DIAL software [53], including annotating polar metabolites (metabolomics workflow) and complex lipids (lipidomics workflow). All mass spectra underwent manual investigation, utilizing retention times and mass spectral information from MS1 and MS/MS libraries. To assess the precision of the overall analytical method, a superior quality control (SQC) sample was prepared by pooling QC samples from each matrix to reflect an aggregated metabolite composition. This SQC sample was aliquoted and repeatedly injected after every set of 35 samples (Fig. S1) and used to correct longitudinal signal drifts using locally estimated scatterplot smoothing (LOESS).

Overall analytical precision was evaluated through PCA of the total variance. As shown in the PCA score plot (Fig. S2), the tight clustering of SQC injections indicates minimal residual technical variability. In contrast, metabolomic and lipidomic profiles from different rat samples were more dispersed, with the first two principal components explaining over 42% of the total biological variance. Analysis of the SQC samples revealed that 84% of all annotated metabolites had excellent reproducibility, with relative standard deviations (RSD) below 10%. Furthermore, 99.2% of metabolites showed RSD values below 20%, underscoring the high quality and consistency of the dataset (Table S1). In total, 851 distinct metabolites were annotated and passed the quality control criteria (see Supplementary materials and methods). Coloring the PCA plots by matrices revealed clear biological differences among sample types. Fig. S3 shows samples from the SCN, mPFC, plasma, and feces formed distinct clusters, indicating pronounced metabolic differences in these compartments. In contrast, other tissue samples exhibited greater overlap, reflecting more subtle metabolic variation.

The annotated metabolites were used for subsequent statistical analyses, including JTK_CYCLE, a nonparametric algorithm for detecting rhythmic components in large datasets [51], and the recently introduced dryR algorithm, which assesses differential rhythmicity in time series data [52]. In addition, the metabolites were also included in multivariate analyses such as PCA and PLS-DA to provide an overview of the dataset [54].

As anticipated, complex lipids constituted the majority of reported metabolites (71%) due to their high endogenous content in various biological matrices. Main lipid classes included (lyso-)phosphatidylcholines (LPC, PC), (lyso-)phosphatidylethanolamines (LPE, PE), triacylglycerols (TG), free fatty acids (FA), acylcarnitines (CAR), phosphatidylserines (PS), phosphatidylinositols (PI), phosphatidylglycerols (PG), sphingomyelins (SM), ceramides (Cer), and diacylglycerols (DG). Organic acids, amino acids, modified amino acids, peptides, hydroxy acids, organic oxygen compounds, organoheterocyclic compounds, organic nitrogen compounds, nucleosides, nucleotides, and others were the main components of the polar metabolome.

Furthermore, the RT qPCR method [45, 48] was employed to detect mRNA levels of clock genes in the liver (*Per1*,* Per2*,* Rev-Erbα*,* Cry1*,* Bmal1*,* Clock*), SCN (*Per2*,* Rev-Erbα*,* Bmal1*,* Dbp*,* E4bp4*), pancreas, and dlWAT (*Per2*,* Rev-Erbα*) for further investigation of the rhythmicity during ontogenesis.

### Web application

The dataset for this study comprises 1610 samples, each containing hundreds of annotated metabolites, including polar metabolites and complex lipids. Clock gene data are also available for four matrices: liver, SCN, pancreas, and dlWAT. Consequently, we developed the Circadian Ontogenetic Metabolomics Atlas (COMA), a web-based application for facilitating data visualization and interpretation (https://coma.metabolomics.fgu.cas.cz).

**Fig. 2:**
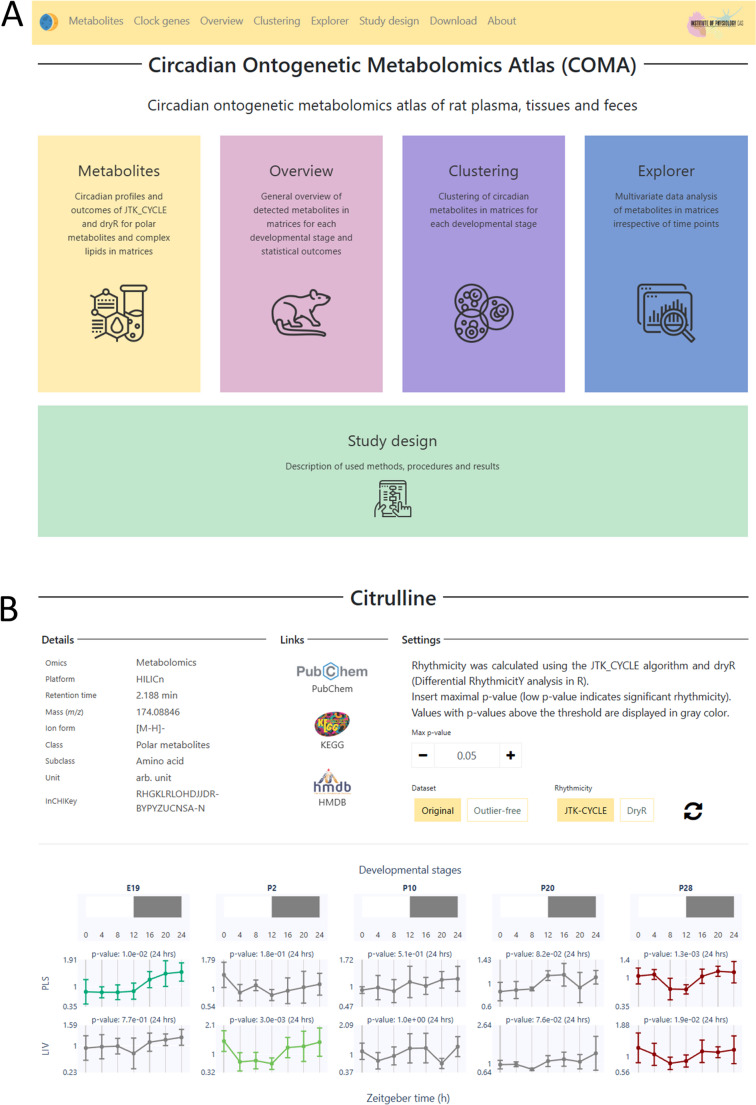
Circadian Ontogenetic Metabolomics Atlas website. (**A**) The initial web page of the Circadian Ontogenetic Metabolomics Atlas; (**B**) example of data visualization of the amino acid citrulline in plasma (PLS) and the liver (LIV) in the “Metabolites” section using the original dataset and JTK_CYCLE algorithm. Oscillating metabolites (*p*<0.05, Bonferroni-adjusted for multiple testing, *ADJ.P*) are colored by default, while non-oscillating metabolites are gray

The atlas is structured into five main sections (Fig. 2A), offering circadian profiles and outcomes of JTK_CYCLE and dryR algorithms for polar metabolites and complex lipids in matrices, a general overview of detected metabolites in matrices for each developmental stage, clustering of circadian metabolites in matrices, multivariate data analysis of metabolites in matrices irrespective of time points, and study design.

The “Metabolites” section overviews all polar metabolites and complex lipids. Users can search for a specific metabolite by name, molecular formula, and InChIKey. Additional search options for complex lipids include the number of carbons and double bonds. The “Metabolomics” section is further divided into “Complex lipids” and “Polar metabolites.” Profiles and outcomes of the JTK_CYCLE and dryR algorithms are displayed when selecting particular metabolites. Data are visualized for each developmental stage (E19, P2, P10, P20, and P28), along with the collected matrix and the calculated adjusted *p*-value (see an example of the amino acid citrulline) in Fig. 2-B. Oscillating metabolites (*p* < 0.05) are colored by default, while non-oscillating metabolites are gray. Users can adjust the *p*-value threshold according to their preferences. Results are available to the entire dataset without outlier detection (marked as the “Original” dataset) and after removing outliers based on the median value and the absolute deviation from the median, using upper and lower limits determined 4 times the median absolute deviation (marked as the “Outlier-free” dataset). Changes take effect after clicking the “Refresh plot” button, enabling users to compare the outcomes of both data pretreatment approaches.

The “Overview” section provides a general overview of the composition of the analyzed matrices for each developmental stage. A sunburst graph visualizes hierarchical data in concentric circles, displaying the total number of unique annotated metabolites for each matrix and developmental stage, sorted into polar metabolites, complex lipids, and their respective classes. It also presents the total number of circadian metabolites identified using the JTK_CYCLE or dryR algorithms with a significance level of *p* < 0.05. Datasets without and with outlier detection were used, and metabolites with *p*-values above the threshold (0.05) are depicted in gray.

The “Clustering” section introduces categories for each matrix and developmental stage, combining JTK_CYCLE (*p* < 0.05) [51] and fuzzy c-means clustering [40, 55]. Six key categories indicate metabolite clusters with specific temporal dynamics based on the initial evaluation of each developmental stage and matrix, and considering typical clusters reported in the literature [40, 56]. The line width and opacity on each graph indicate the number of metabolites.

The “Explorer” section encompasses a statistical analysis of analyzed matrices, irrespective of Zeitgeber times. Matrices are categorized into digestive (liver, pancreas, stomach, small intestine), excretory (kidney, feces), respiratory (lungs), endocrine (spleen, isWAT, dlWAT, iBAT), muscular (gastrocnemius skeletal muscle), cardiovascular (plasma, heart), and nervous (SCN, mPFC) systems. Results of multivariate data analysis are presented using unsupervised PCA to visualize the first two principal components through scores and loadings plots. Supervised PLS-DA is displayed with the first two components in scores and loadings plot formats, featuring a color-based gradient indicating VIP scores for all metabolites. In both PCA and PLS-DA, samples in score plots are color-coded based on the developmental stage. A sunburst graph offers a comprehensive overview of annotated metabolites in a specific matrix. Box plots are also presented for each metabolite sorted by VIP, and clicking on another metabolite updates the corresponding box plot.

The “Study design” section summarizes experimental conditions, including details about experiments involving animals, the methodology employed, and abbreviations utilized throughout the atlas.

Clock genes are accessible using the upper panel, and the data are visualized similarly to that of metabolites. This panel also includes a download section where users can download the whole data set, including metadata.

### Exploring the circadian ontogenetic metabolomics atlas

In the following examples, we provide a snapshot of the results of the Circadian Ontogenetic Metabolomics Atlas, including the investigation of circadian metabolites in each matrix and developmental stage, the clustering of metabolites, and finally, general exploration using multivariate data analysis.

**Fig. 3:**
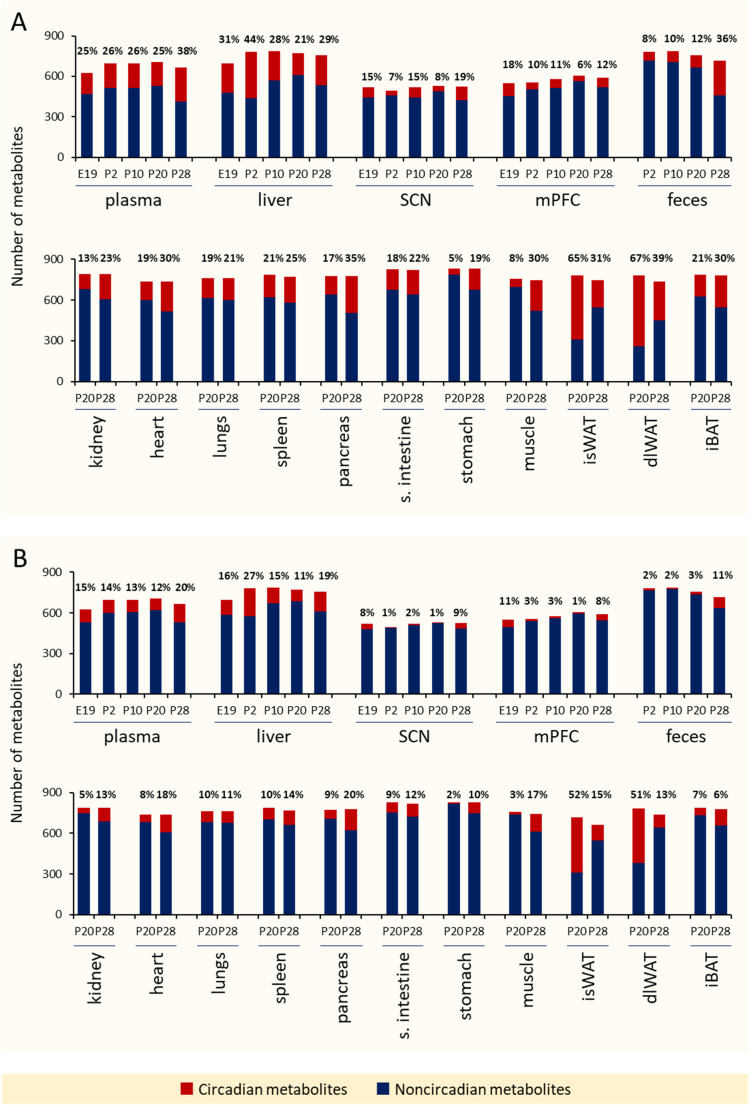
Overview of the total number and percentage of circadian metabolites by matrix and developmental stage. The original dataset (without outlier analysis) was processed using (**A**) JTK_CYCLE, considering metabolites with Bonferroni-adjusted *p*<0.05 (*ADJ.P*) as statistically significant, and (**B**) dryR, considering metabolites with Benjamini–Hochberg-adjusted *p*<0.05 (*padj*) as statistically significant

The results from applying the JTK_CYCLE and dryR algorithms to identify and characterize cycling metabolites are shown in Fig. 3. The figure provides an overview of the percentage of circadian metabolites, defined as those with adjusted *p*-values (< 0.05; *ADJ.P* for JTK_CYCLE and *padj* for dryR) and a period (PER) of 24 h. Across matrices and developmental stages, the average percentage of oscillating metabolites was approximately 24% with JTK_CYCLE and 12% with dryR. Stomach tissue, SCN, and mPFC were identified with the lowest number of circadian metabolites, while dlWAT, isWAT, liver, and plasma had the highest number of circadian metabolites.

**Fig. 4:**
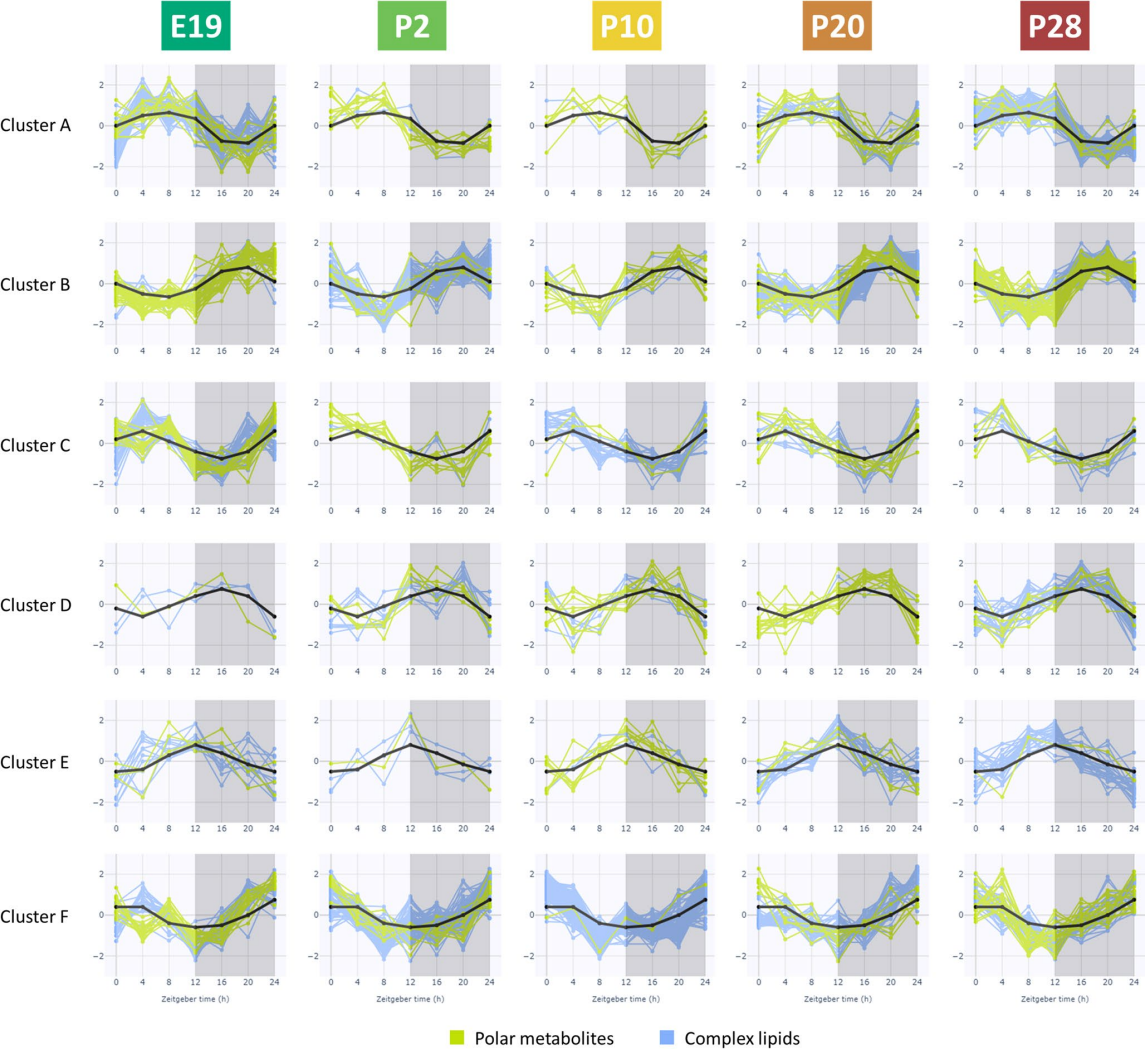
Cluster analysis of plasma samples by developmental stage. Six clustering categories are shown. Only polar metabolites and complex lipids with significant circadian oscillation (JTK_CYCLE, *ADJ.P*<0.05) were included in the analysis

An example of the clustering of metabolites is provided in Fig. 4 on plasma samples for all five developmental stages, illustrating commonalities in their temporal dynamics. These categories of the clusters can be characterized as follows:

(A) Metabolites with a gradual increase during the daytime inactive phase, reaching a maximum of around 4–8 h, followed by a decrease with a minimum at 16–20 h;
(B) Metabolites with a gradual decrease during the daytime inactive phase, reaching a minimum of around 4–8 h, followed by an increase with a maximum at 16–20 h;
(C) Metabolites exhibiting a gradual increase during the daytime inactive phase and a rapid decrease when feeding starts at night;
(D) Metabolites rapidly decreasing at the onset of the inactive phase, rising upon feeding until plateauing in the middle of the night;
(E) Metabolites exhibiting a gradual increase during the daytime inactive phase and a rapid decrease when feeding starts at night;
(F) Metabolites with a shallower decline during the inactive phase, a temporary decrease at the onset of feeding, followed by an increase 2 h after feeding begins.

For the E19 developmental stage, cluster A contained the highest number of metabolites (32%) within the clusters, whereas for P2 and P10, metabolites in cluster F dominated (45% and 65%, respectively). In the late developmental stage P20, cluster B exhibited the highest number of metabolites (42%), while metabolites in cluster A dominated the developmental stage P28 (40%). These data suggest that metabolite profiles undergo significant changes during ontogenesis.

Since the lipidome constituted the largest proportion of reported metabolites, we also implemented downloaded lists of lipids formatted for a recently introduced web-based tool called Lipid Over-Representation Analysis (LORA) [57]. LORA determines whether a priori-defined set of lipids is more present (over-represented) in a subset of lipids than would be expected by chance. A list of query lipids can be downloaded from the “Cluster” section for each matrix and developmental stage. Additionally, the entire reference lipidome can be obtained from the “Download” section (lipidome_universe.csv) and uploaded to the LORA tool at https://lora.metabolomics.fgu.cas.cz. Fig. S4 illustrates an example of plasma at the P28 developmental stage for cluster A, revealing 127 statistically significant metabolites, most of which (*n* = 120) are complex lipids. The UpSet plot helps identify the main structural features of enriched lipids and highlights important lipids in a graphical representation, particularly TGs containing 10:0, 12:0, and 14:0 fatty acyl chains. Using the cardinality bar plot, the cluster with the highest term intersection size (*n* = 18) contained TGs with a 10:0 fatty acyl chain. The second cluster contained 14 lipid species with TGs containing a 12:0 fatty acyl chain.

**Fig. 5:**
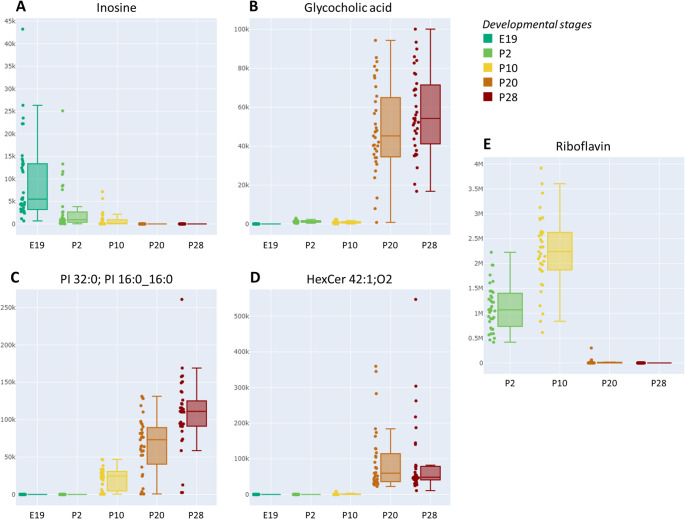
Box plots for the most discriminating metabolites in (**A**) plasma, (**B**) liver, (**C**) SCN, (**D**) mPFC, and (**E**) feces at different developmental stages based on PLS-DA and VIP

Furthermore, the atlas can also be used for a more general audience using the “Explorer” function. Using PLS-DA and VIP scores, it became apparent that distinct metabolites were characteristic of the analyzed matrices. For instance, in plasma, inosine (a nucleoside) dominated during the fetal E19 stage, continuously declining in postnatal stages. In the liver, glycocholic acid (a bile acid) was completely absent during fetal and early postnatal P2 and P10 stages and dominated in the P20 and P28 stages (Fig. 5A, B). In the SCN, the lipid PI 32:0 was also absent during the fetal and early postnatal P2 stage and increased over the P10, P20, and P28 stages (Fig. 5C). Similarly, HexCer 42:1;O2 was detected at higher levels in mPFC during postnatal P20/P28 stages (Fig. 5D). In feces, the vitamin riboflavin dominated during the early postnatal P2/P10 stages and was almost absent in the P20/P28 stages (Fig. 5E). These examples also show that the metabolome undergoes dramatic changes from the fetal to late postnatal developmental stages. For the rest of the matrices collected at the P20 and P28 postnatal stages, differences were observed in a way that a few metabolites occurred only in one developmental stage or differed in their intensities for most of them. These data also underscore the critical role of sampling time in metabolomics analyses, as the intensity of specific metabolites can vary significantly based on the time of collection and developmental stage. Metabolites are influenced by circadian rhythms, which govern biological processes and fluctuate throughout the day and night. This timing-dependent variation can impact the detection and quantification of metabolites, potentially leading to biased or inaccurate interpretations if sampling is inconsistent. Using animals within the same developmental stage, or “age-matched” subjects, alongside standardized sampling times, is essential to minimize variability and improve sample comparability.

Users can also explore the analysis of clock genes. For instance, *Bmal1*, *Per2*, *Nr1d1*, and *Dbp*, in the SCN, revealed shallow rhythms at E19, followed by initiation at P2. Rhythms increased at P10, and high amplitude was observed at the P20 and P28 developmental stages (Fig. S5), as observed before in fetal E20 and early postnatal P1, P2 [58], and P10 [59] developmental stages, as well as in adult rats [11].

### Potential for the discovery of novel metabolites

Hundreds of unique metabolites can usually be annotated while combining multiple platforms during untargeted metabolomics and lipidomics analyses. However, many more signals can be detected, characterized by retention time and *m*/*z* (i.e., molecular features) [60]. The raw LC–MS instrumental files provided with this atlas can be reprocessed, including updated MS/MS libraries, to increase the annotation rate further, or researchers can focus on the structural elucidation of unknown metabolites.

**Fig. 6:**
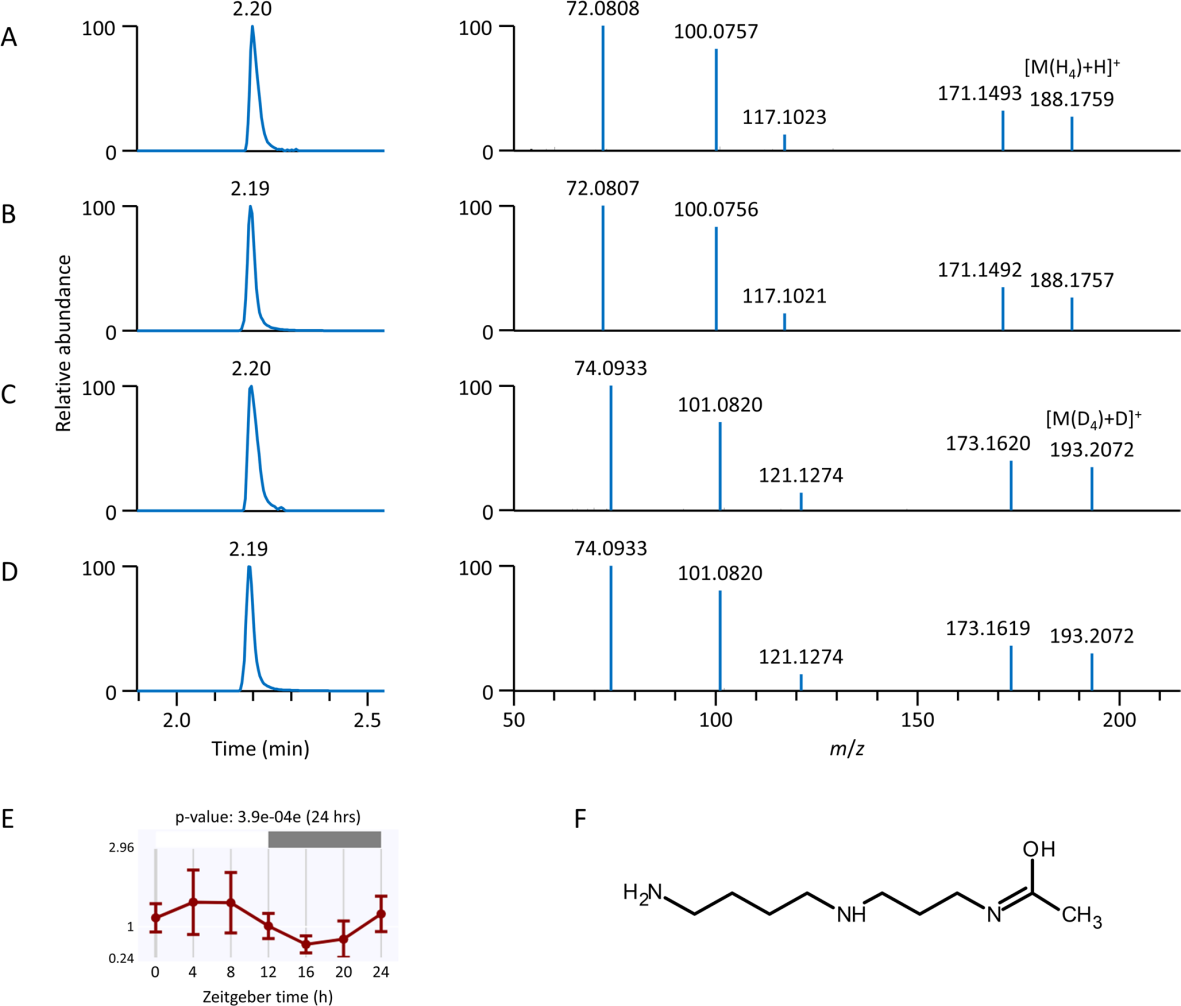
Structure elucidation of an unknown metabolite. (A, B) Extracted ion chromatograms (EICs) and MS/MS spectra of *N*^1^-acetylspermidine in (**A**) rat gastrocnemius skeletal muscle and (**B**) analytical standard analysis under conventional HILIC–MS with the EIC at *m*/*z* 188.1757 displayed corresponding to [M(H_4_)+H]^+^. (C, D) EICs and MS/MS spectra of *N*^1^-acetylspermidine in (**C**) rat gastrocnemius skeletal muscle and (**D**) analytical standard analysis under HILIC–HDX-MS with the EIC at *m*/*z* 193.2071 displayed corresponding to [M(D_4_)+D]^+^. (**E**) Circadian rhythmicity of *N*^1^-acetylspermidine in gastrocnemius skeletal muscle at the P28 developmental stage, calculated using JTK_CYCLE with a Bonferroni-adjusted *p*-value (*ADJ.P*). (**F**) Structure of *N*^1^-acetylspermidine

For example, we initially observed an unknown with a retention time of 2.2 min and *m*/*z* 188.1757 without any positive spectral match when using the combined MS/MS libraries during the HILIC platform in positive ion mode. Submitting MS1 isotopic ions and the MS/MS spectrum from MS-DIAL [53] to the MS-FINDER [61] software for structural elucidation provided over 100 possible unique structures. Since focusing on many structures would be challenging, we utilized the potential of hydrogen/deuterium exchange mass spectrometry (HDX-MS). Thus, for the HILIC–MS metabolomics platform, which uses acetonitrile/water (95:5) and water as mobile phases, both with ammonium formate and formic acid, water and mobile-phase modifiers were replaced by their deuterated forms (D_2_O, ammonium formate-*d*_5_, formic acid-*d*_2_). This setup permitted complete hydrogen/deuterium exchange and an additional filter from hydrogen/deuterium exchange mass spectrometry (HDX-MS) [62], reducing the number of potential candidates to one-fifth due to four labile hydrogens in the molecule as determined by a mass spectrometer. The subsequent analysis of the standard of *N*^1^-acetylspermidine confirmed the identity of this unknown based on retention time, MS1, and MS/MS spectra, including the number of exchangeable hydrogens in the parent ion and fragments (Fig. 6) when using unlabeled mobile-phase modifiers and labeled counterparts. Furthermore, *N*^1^-acetylspermidine exhibited circadian rhythmicity in different tissues at late developmental stages (P20, P28), as shown in the example of gastrocnemius skeletal muscle (Fig. 6).

## Discussion

### Selection of matrices and developmental stages

We investigated the development of the circadian metabolome in the rat brain (SCN, mPFC) and periphery (liver, plasma) across five different developmental stages. Furthermore, feces were studied at four developmental stages (P2, P10, P20, P28), while the other 11 tissues (kidney, heart, lung, spleen, pancreas, small intestine (jejunum), stomach, gastrocnemius skeletal muscle, isWAT, dlWAT, and iBAT) were examined at late developmental stages (P20, P28). As a well-established approach, clock gene expression was analyzed in the SCN, liver, pancreas, and ldWAT.

The SCN and mPFC were selected due to their significant developmental changes during both embryonic and postnatal stages. The SCN, as the central circadian clock, plays a crucial role in developing the circadian system [10]. The liver, where approximately 10% of the transcriptome exhibits rhythmic expression, regulates glucose, lipid, and nutrient homeostasis, as well as bile acid synthesis and metabolism. Plasma, acting as a vital link between peripheral tissues [63], was also included in the study. Feces were included because the circadian system influences various gastrointestinal processes and is influenced by feeding time [64].

Clocks, similar to those found in SCN neurons, are present in peripheral tissues [65–67]. For example, various renal functions exhibit circadian rhythms, such as renal plasma flow and glomerular filtration rate. Alterations in the circadian rhythm of renal functions are associated with developing hypertension, chronic kidney disease, renal fibrosis, and kidney stones [68]. Moreover, evidence indicates a close relationship between intrinsic circadian clocks and cardiovascular functions. Well-known circadian rhythms include diurnal changes in blood pressure and heart rate. Animal models and epidemiological studies provide strong evidence that the disruption of circadian rhythms is a significant risk factor for many cardiovascular diseases [69]. Circadian rhythms play an important role in regulating the digestive systems of many organisms. Cell proliferation, migration, differentiation, and even structure vary as a function of the time of day in various digestive organs (such as the liver, pancreas, and small intestine) and cell types, leading to regionally specific temporal variations in protein and gene expression [70, 71]. A link also exists between the circadian clock and rhythmic immune functions. The spleen, lymph nodes, and peritoneal macrophages contain an autonomously intrinsic circadian clockwork [72]. In mammals, pulmonary function follows day–night patterns. Hence, the molecular clock function in lung cells may function as a biomarker for disease severity and exacerbations or for evaluating the effectiveness of chronotherapy in disease management [73]. The cell-intrinsic clock machinery in skeletal muscle could be critical for whole-body metabolic homeostasis [74]. Last but not least, the circadian clock controls different aspects of lipid metabolism in WAT and BAT, including lipolysis, lipogenesis, and BAT thermogenesis [75].

The chosen developmental stages represent key milestones in rat development [10, 11]. At E19, neurogenesis is completed, although the morphological maturation of SCN neurons has not yet been concluded. In the early postnatal period (P2), substantial reorganization and functional specialization occur in the rat SCN, forming various cell subpopulations. By postnatal stage P10, the SCN clock is fully developed, while the pups’ eyes remain closed, and they are still entirely dependent on maternal care. The period between P10 and P20/P28 marks a developmental phase during which pups gain sight, experience a gradual decline in breast milk intake, and initiate solid food consumption at night, influencing the phases of their peripheral clocks [76]. A comparison between postnatal stages P20 and P28 was included to generate evidence for arguments in favor of or against premature weaning, typically performed at P21. Some studies suggest that later stages might be more appropriate [77].

### Experimental considerations

When interpreting circadian metabolomics data, it is essential to account for various experimental aspects that may influence the detection and characterization of rhythmic metabolites [78]. These variables include diet, feeding schedule, light–dark cycle, and sampling time [78]. For instance, Dyar et al. [38] demonstrated that, in mice, a chow diet maintained strong temporal coherence of metabolites within and across tissues, whereas a high-fat diet disrupted this coherence and rewired circadian metabolism. Additionally, under *ad libitum* (free-feeding) conditions, rodents (particularly nocturnal species like rats and mice) often engage in sporadic daytime feeding or “snacking” behavior, which can influence both metabolomic and transcriptomic profiles [52, 79].

Although laboratory conditions provide tight control of light and feeding, real-world factors like shift work and artificial lighting can significantly disrupt circadian rhythms, affecting metabolic oscillation amplitude, phase, and robustness [78]. Future studies incorporating dynamic environmental conditions or leveraging human cohort data with diverse lifestyle factors could enhance our understanding of how external cues influence circadian metabolic regulation.

In addition to experimental factors, statistical modeling also plays a crucial role. In our analysis, we applied the well-established JTK_CYCLE algorithm [51] alongside the recently introduced dryR model [52]. Across matrices and developmental stages, the average percentage of oscillating metabolites was approximately 24% with JTK_CYCLE and 12% with dryR. This two-fold difference can be attributed to the underlying assumptions and sensitivity of the respective methods. JTK_CYCLE is a nonparametric algorithm optimized for detecting robust, sinusoidal rhythms with fixed periods (e.g., 24 h), making it particularly sensitive to clear, consistent oscillations. In contrast, dryR uses linear mixed-effects regression to model rhythmicity, accounting for amplitude and phase variability across conditions. This makes it more conservative and better suited to identifying context-specific or differential rhythmicity. Consequently, dryR may detect fewer circadian metabolites but potentially capture more biologically nuanced patterns. In JTK_CYCLE, adjusted *p*-values (*ADJ.P*) reflect multiple testing correction, with *ADJ.P* < 0.05 commonly used to define circadian regulation [38]. However, it is often useful to compare multiple *ADJ.P* thresholds and empirically assess the biological plausibility of rhythmic patterns [80].

The disparity in circadian detection between methods highlights how methodological choices can influence the interpretation of rhythmicity in high-dimensional omics datasets. Nonetheless, the percentage of circadian metabolites reported here aligns with recent findings from a mouse circadian metabolome atlas, which showed that 20–50% of metabolites across various tissues (SCN, mPFC, muscle, BAT, WAT, liver, sperm) and serum displayed 24-hour oscillations regardless of diet [38].

Developmentally, except for isWAT and dlWAT, the P20 stage exhibited fewer circadian metabolites than P28 across all matrices (Fig. 3**)**. Moreover, using the dryR model to assess rhythmicity across late developmental stages (P20 and P28) in parallel, we observed a loss of rhythm in approximately 12% of metabolites, a gain of rhythm in 13%, stable rhythmicity in 12%, and altered rhythmicity in 2% (Table S2). The transitional period between P20 and P28 encompasses physiological and behavioral changes associated with weaning, likely influencing tissue metabolite rhythmicity. At P20, pups are still partially reliant on maternal milk, whereas by P28, they are typically fully weaned and consuming solid chow, establishing circadian-regulated feeding behaviors more akin to adult patterns. For instance, using the “Explorer” function in COMA, we identified characteristic temporal profiles of plasma TGs containing short-chain saturated fatty acids. These TGs are synthesized *de novo* from maternal milk enriched in saturated fatty acids [81]. Their abundance peaks at P10, declines at P20, and drops to very low levels by P28, consistent with the weaning transition (Fig. S6**)**.

As noted, the metabolome shows dramatic changes from fetal to late postnatal stages, as highlighted by the most discriminating metabolites in PLS-DA and VIP (Fig. 5) through the “Explorer” function. Purine metabolites, such as inosine, hypoxanthine, and adenosine, progressively decline from E19 to P28 (Fig. S7). This decline likely reflects a shift from high purine turnover during rapid growth and nucleic acid synthesis to lower metabolic demands in more mature stages. These metabolites, associated with hypoxia and energetic stress, suggest a developmental maturation from rapid growth to homeostasis [82]. HexCer 42:1;O2 and related species (HexCer 40:1;O2 and HexCer 41:1;O2) increased markedly in the mPFC from E19 to P28 (Fig. 5D, Fig. S8), aligning with oligodendrocyte maturation and myelination. These glycosphingolipids support membrane structure and signaling, and their rise reflects growing myelin synthesis and membrane remodeling during postnatal cortical development [83]. Glycocholic acid, a glycine-conjugated primary bile acid, exhibited a marked increase in rat liver from early postnatal to weaning stages (Fig. 5B). This dramatic shift likely reflects the maturation of hepatic bile acid synthesis pathways and the onset of enterohepatic circulation, which become fully functional around weaning [84]. In the prenatal and early postnatal period (E19–P10), the fetal liver has limited bile acid production, and maternal supply dominates. By P20–P28, the weaning transition drives increased dietary fat intake, requiring enhanced bile acid secretion for digestion and absorption. Riboflavin levels in rat feces were high at early postnatal stages (P2 and P10) but declined sharply by P20 and were nearly undetectable at P28 (Fig. 5E). This likely reflects excess riboflavin from maternal milk, immature vitamin absorption, and limited microbial utilization in early life [85]. As the gut matures and diet shifts during weaning, fecal riboflavin levels decrease accordingly.

### An open-access resource for circadian metabolomics

Understanding the circadian regulation of metabolism requires comprehensive datasets that capture metabolite rhythms across tissues and developmental stages. However, bioinformatics resources offering circadian metabolite profiles in animals remain limited, and detailed information on tissue-specific metabolite composition is scarce. Currently, the only available platform is the CircadiOmics web portal (https://circadiomics.igb.uci.edu), a repository and analytical tool for circadian omics data (transcriptomic, proteomic, and metabolomic) [86]. It includes metabolomics datasets primarily from mouse studies, covering plasma/serum and various tissues (e.g., brain, liver, muscle, adipose tissue) under different experimental conditions (e.g., chow vs. high-fat diet, exercise, wild-type vs. knockout models). While users can select datasets and request visualizations and statistical analyses (using BIO_CYCLE.2 and JTK_CYCLE), there is no comprehensive browser or complete list of metabolites for individual studies. Users must first download the metabolite list from the original publication, typically from supplementary materials, before querying the web portal to search for a particular metabolite. Additionally, the portal does not include datasets focused on developmental biology, and raw LC–MS data files are not available for reprocessing.

To address this gap, we aimed to create an open-access dataset covering the circadian metabolome of multiple rat matrices (plasma, tissues, feces), ensuring the data are easily accessible and readily usable by the scientific community. We employed a combined extraction method for polar metabolites and complex lipids, followed by a multiplatform LC–MS-based approach, expanding the breadth and scope of covered metabolites. This enables a more thorough examination of circadian metabolic dynamics across different developmental stages and tissue types.

By sharing raw LC–MS instrumental files, we enable retrospective data mining, allowing for additional annotation of unknowns through MS/MS library searches and the potential discovery of novel metabolites. This study acquired LC–MS data in data-dependent acquisition (DDA) mode for all samples. We also employed iterative exclusion to remove background and previously selected high-abundance precursor ions, thereby increasing the likelihood of capturing MS/MS spectra from less abundant precursors. Additionally, LC–HDX-MS supported structural elucidation by reducing false positives using the number of exchangeable hydrogens to refine molecular formulas and distinguish isobaric species [87].

Adherence to the FAIR guiding principles ensures that metabolomics data generated during our analyses are Findable, Accessible, Interoperable, and Reusable (FAIR) [88]. This involves making raw instrumental LC–MS files publicly available, including samples, blanks, and quality control samples. Sharing data in a repository ensures accessibility to the scientific community. The FAIR principles also emphasize the importance of using harmonized formats to make data interoperable and ensure that data remains reusable with long-term validity, independent of time. By following these principles, we contribute to the reproducibility and transparency of metabolomics research.

### Limitations of the study

This study used Wistar: Han rats, which may limit the generalizability of the findings to other species, including humans. Comparative analyses with genetically modified models could offer deeper insight into the role of specific genetic factors in shaping circadian metabolic rhythms. In addition, the utility of circadian metabolite atlases could be further enhanced by integrating non-sacrificial longitudinal sampling of biofluids (e.g., urine, blood) and feces under varied light and feeding conditions. Such an approach would allow for intra-individual tracking of metabolic rhythms, reduce inter-animal variability, and provide a more ecologically valid and clinically relevant picture of circadian metabolite dynamics.

From a technical perspective, LC–MS-based metabolomics is limited by metabolite coverage, ionization efficiency, and semi-quantitative reproducibility across diverse sample types [87]. Our combined extraction and LC–MS platforms [47] were optimized for high- and medium-abundance metabolites but may underrepresent low-abundance species. Detecting these would require specialized extraction protocols or alternative platforms with higher sensitivity. Although MS/MS data were acquired for all study samples, many metabolites remain unannotated due to limitations in current metabolomics databases. Expanding spectral libraries and incorporating advanced structural elucidation techniques could improve annotation rates [89].

## Supplementary Information

Below is the link to the electronic supplementary material.

Supplementary file1 (PDF 1.42 MB)

Supplementary file2 (XLSX 75.7 KB)

Supplementary file3 (XLXS 1.44 MB)

## Data Availability

The Circadian Ontogenetic Metabolomics Atlas (COMA) is accessible at https://coma.metabolomics.fgu.cas.cz, and includes processed data used for circadian analysis across various developmental stages. Raw files in mzXML format for all LC–MS platforms are available at 10.5281/zenodo.13843399 and also include metadata for study samples, method blanks, QC, and serial dilution samples for each LC–MS platform.
