# Supplementary material for: Circadian ontogenetic metabolomics atlas: an interactive resource with insights from rat plasma, tissues, and feces
Source: Cell Mol Life Sci. 2025 Jun 28;82(1):264. doi: 10.1007/s00018-025-05783-w (PMC12206216; doi:10.1007/s00018-025-05783-w)
Supplement: Supplementary file 1 — Supplementary file1 (PDF 1.42 MB) [file 18_2025_5783_MOESM1_ESM.pdf]

Supplementary information

Circadian ontogenetic metabolomics atlas: an interactive resource with insights from rat plasma, tissues, and feces

Lucie Rudl Kulhava,[1,†] Pavel Houdek,[1,†] Michaela Novakova,[1,†] Jiri Hricko,[1] Michaela Paucova,[1] Ondrej Kuda,[1] Martin Sladek,[1] Oliver Fiehn,[2] Alena Sumova,[1,‡,*] Tomas Cajka[1,‡,*]

[1] Institute of Physiology of the Czech Academy of Sciences, Videnska 1083, Prague, 14200, Czech Republic

[2] University of California, Davis, 451 Health Sciences Drive, Davis, CA, 95616, United States

[†] These authors contributed equally

[‡] Senior authors

[*] E-mails: Alena Sumova; alena.sumova@fgu.cas.cz and Tomas Cajka; tomas.cajka@fgu.cas.cz

DOI: 10.1007/s00018-025-05783-w

Supplementary materials and methods

Figures S1–S8

Supplementary materials and methods

Reagents for LC–MS-based metabolomics

For sample extraction, methanol (J.T.Baker, catalog no. 9822), methyl *tert*-butyl ether (Honeywell, catalog no. 34875), and water (VWR, catalog no. 83645.320) were used. LC–MS-grade solvents for mobile phases included acetonitrile (Honeywell, catalog no. 34967), methanol (J.T.Baker, catalog no. 9822), water (VWR, catalog no. 83645.320), and isopropanol (Supelco, catalog no. 1027812500) [1]. Mobile phase modifiers, such as ammonium formate (Supelco, catalog no. 70221), ammonium acetate (Sigma–Aldrich, catalog no. A7330), formic acid (VWR, catalog no. 84865.260), and acetic acid (VWR, catalog no. 84874.180), were also of LC–MS-grade quality [1]. For HILIC–HDX-MS experiments, ammonium formate-d_5 (Merck, catalog no. 795119), formic acid-d_2 (CDN Isotopes, catalog no. DLM-286-PK), deuterium oxide (Merck, catalog no. 617385), and N^1-acetylspermidine (Cayman Chemical, catalog no. 9001535) were used [2]. Internal standards were obtained from Avanti, Cayman Chemical, CDN Isotopes, Merck, Sigma–Aldrich, and Supelco.

Sample extraction

For the sample extraction, a biphasic solvent system of methanol, methyl *tert*-butyl ether (MTBE), and water was used to isolate complex lipids, polar metabolites, and exposome compounds (LIMeX workflow) [1, 3-5]. An aliquot of 25 µL was used for plasma, and approximately 20 mg was used for tissues, with exceptions for mPFC (E19: 2–10 mg; P2–P28: 10 mg), feces (E19: 1.8–5.2 mg; P2–P28: 10 mg), and SCN (dissected SCN was isolated by a pre-cooled microbiopsy punch, resulting in a cylindrical sample of SCN-containing frozen tissue with a 0.3 mm diameter and approximately 0.4 mm height).

Plasma. A volume of 165 µL methanol containing internal standards (CAR 14:0-d_9, CAR 16:0-d_3, CAR 18:0-d_3, Cer d18:1/17:0, cholesterol-d_7, CL 16:0/16:0/16:0/16:0, DG 12:0/12:0/0:0, DG 18:1/0:0/18:1-d_5, DG 18:1/2:0/0:0, Hex-Cer d18:1/17:0, LPC 17:1, LPE 17:1, LPG 17:1, LPS 17:1, MG 17:0/0:0/0:0, oleic acid-d_9, PC 15:0/18:1-d_7, PE 17:0/17:0, PG 17:0/17:0, PI 15:0/18:1-d_7, PS 17:0/17:0, SM d18:1/17:0, sphingosine d17:1, TG 17:0/17:1/17:0-d_5, TG 20:0/20:1/20:0-d_5) was added to plasma aliquot and shaken (30 s) followed by addition of 600 µL of MTBE with internal standard (CE 22:1) and shaking (30 s). Then, 165 µL of 10% methanol containing also internal standards (3-hydroxybutyric acid-d_4, acetylcholine-d_4, alanine($^{13}C_3$; ^{15}N), arginine($^{13}C_6$; $^{15}N_4$), aspartic acid($^{13}C_4$; ^{15}N), betaine-d_9, butyrobetaine-d_9, caffeine-d_9, carnitine-d_9, CAR 2:0-d_3, CAR 3:0-d_3, CAR 4:0-d_3, CAR 6:0-d_3, CAR 8:0-d_3, CAR 10:0-d_3, CAR 12:0-d_9, choline-d_9, citrulline-d_4, cotinine-d_3, creatine-d_3, creatinine-d_3, cystine($^{13}C_6$; $^{15}N_2$), glucose-d_7, glutamic acid($^{13}C_5$; ^{15}N), glycine($^{13}C_2$; ^{15}N), histidine($^{13}C_6$; $^{15}N_3$), isoleucine($^{13}C_6$; ^{15}N), leucine($^{13}C_6$; ^{15}N), lysine($^{13}C_6$; $^{15}N_2$), metformin-d_6, methionine($^{13}C_5$; ^{15}N), *N*-methylnicotinamide-d_4, ornithine-d_6, phenylalanine($^{13}C_9$; ^{15}N), proline($^{13}C_5$; ^{15}N), serine($^{13}C_3$; ^{15}N), succinic acid-d_4, threonine($^{13}C_4$; ^{15}N), trimethylamine *N*-oxide-d_9, tyrosine($^{13}C_9$; ^{15}N), valine($^{13}C_5$; ^{15}N)) was added, the tubes were vortexed (10 s) and centrifuged (16,000 rpm, 5 min, 4 °C).

The first aliquot of 60 µL of the bottom phase was collected and evaporated to analyze polar metabolites. The dry plasma extracts were resuspended in 60 µL of an acetonitrile/water (4:1) mixture with two internal standards (12-[[(cyclohexylamino)carbonyl]amino]-dodecanoic acid (CUDA) and Val-Tyr-Val), shaken (30 s), centrifuged (16,000 rpm, 5 min, 4 °C), and analyzed using the HILIC metabolomics platforms in positive and negative electrospray ionization (ESI) mode. The second 60 µL aliquot of the bottom phase was mixed with 180 µL of an isopropanol/acetonitrile (1:1) mixture, shaken (30 s), centrifuged (16,000 rpm, 5 min, 4 °C), and the supernatant was evaporated. The dry plasma extracts were resuspended in 5% methanol/0.2% formic acid containing two internal standards (CUDA and Val-Tyr-Val), shaken (30 s), centrifuged (16,000 rpm, 5 min, 4 °C), and analyzed using the RPLC metabolomics platform in positive and negative ESI mode. An aliquot of 100 µL of the upper phase was collected and evaporated to analyze complex lipids. The dry extracts were resuspended in 100 µL methanol containing internal standards (arachidonic acid-d_{11}, CAR 24:0-d_4, CE 18:1-d_7, Cer d18:1/10:0, CUDA, DG 15:0/18:1-d_7, GlcCer d18:1/12:0, LPC 18:1-d_7, LPE 18:1-d_7, LPG 13:0, LPI 17:1, LPS 13:0, MG 18:1-d_7, PC 13:0/13:0, PE 15:0/18:1-d_7, PG 15:0/18:1-d_7, PI 8:0/8:0, PS 14:0/14:0, sGalCer d18:1/12:0, SM d18:1/12:0, sphingosine 18:1-d_7, TG 15:0/18:1/15:0-d_7), shaken (30 s), centrifuged (16,000 rpm, 5 min, 4 °C), and analyzed using the RPLC lipidomics platform in positive and negative ESI mode.

Tissues and feces. Tissue and feces sample aliquots were homogenized with 275 µL methanol containing internal standards using a grinder (frequency 1/s: 30, 1.5 min). Then, 1 mL of MTBE with internal standard was added, and the tubes were shaken (30 s). A volume of 275 µL 10% methanol was added, and after vortexing (10 s), the tubes were centrifuged (16,000 rpm, 5 min, 4 °C). Analysis of polar metabolites was performed similarly to that of plasma samples. For complex lipid analysis, 100 µL aliquots were collected for feces and tissues except for mPFC and SCN; in this case, 400 µL aliquots were taken to increase the lipidome coverage. After solvent evaporation, the dry extracts were resuspended in 100 µL (feces), 300 µL (non-fat tissues), or 50 µL (mPFC, SCN) methanol containing a mixture of internal standards, shaken (30 s), centrifuged (16,000 rpm, 5 min, 4 °C), and analyzed using the RPLC lipidomics platform in positive and negative ESI mode.

Adipose tissues. Adipose tissue sample aliquots were homogenized with 275 µL methanol containing internal standards using a grinder (frequency 1/s: 30, 1.5 min). Then, 1 mL of MTBE with internal standard was added, and the tubes were shaken (30 s). A volume of 275 µL 10% methanol was added, and after vortexing (10 s), the tubes were centrifuged (16,000 rpm, 5 min, 4 °C). Analysis of polar metabolites was performed similarly to that of plasma samples. For analysis of minor complex lipids, 100 µL of the upper organic phase was collected, evaporated, resuspended using 100 µL 90% methanol with an internal standard (CUDA), shaken (30 s), centrifuged (16,000 rpm, 5 min, 4 °C), and analyzed using the RPLC lipidomics platform in positive and negative ESI mode. For analysis of abundant triacylglycerols, 10 µL aliquot of the upper organic phase was collected, evaporated, resuspended using 1 mL methanol containing a mixture of internal standards, shaken (30 s), centrifuged (16,000 rpm, 5 min, 4 °C), and analyzed using the RPLC lipidomics platform in positive ESI mode.

LC–MS analysis

For LC–MS analysis, a Vanquish UHPLC system (Thermo Fisher Scientific), a heated electrospray ionization (HESI-II) probe (Thermo Fisher Scientific), and a Q Exactive Plus mass spectrometer (Thermo Fisher Scientific) were used [4].

Polar metabolites. For the separation of polar metabolites based on the HILIC mechanism, the following conditions were used: an ACQUITY Premier BEH Amide column (50 mm length × 2.1 mm i.d.; 1.7 µm particle size) with a VanGuard FIT cartridge (5 mm length × 2.1 mm i.d.; 1.7 µm particle size) (Waters, catalog no. 186010380); column compartment temperature, 45 °C; column flow rate, 0.8 mL/min; mobile phase A, water with 10 mM ammonium formate and 0.125% formic acid; mobile phase B, acetonitrile/water (95:5) with 10 mM ammonium formate and 0.125% formic acid; gradient run, 0 min 100% B, 0–0.5 min 100% B, 0.5–2.0 min from 100% to 70% B, 2.0–2.6 min from 70% to 30% B, 2.6–3.2 min from 30% to 100% B, 3.2–3.4 min 100% B +1 min preinjection steps; injection volumes, 0.3–1 µL (based on the matrix) in ESI(+) and 5 µL in ESI(−) [4]. For the HILIC–HDX-MS platform, the conditions were identical, except for using mobile phase A consisting of D_2O with 10 mM ammonium formate-d_5 and 0.125% formic acid-d_2, and mobile phase B made from acetonitrile/D_2O (95:5) with 7.5 mM ammonium formate-d_5 and 0.125% formic acid-d_2 [2].

For the separation of polar metabolites based on the RPLC mechanism, the following conditions were applied: an ACQUITY Premier HSS T3 column (50 mm length × 2.1 mm i.d.; 1.8 µm particle size) with a VanGuard FIT cartridge (5 mm length × 2.1 mm i.d.; 1.8 µm particle size) (Waters, catalog no. 186009470); column compartment temperature, 45 °C; column flow rate, 0.6 mL/min; mobile phase A, water with 0.2% formic acid; mobile phase B, methanol with 0.1% formic acid; gradient run, 0 min 1% B, 0–0.5 min 1% B, 0.5–2 min from 1% to 60% B, 2–2.3 min from 60% to 99% B, 2.3–2.8 min 99% B, 2.8–2.9 min from 99% to 1% B, 2.9–3.4 min 1% B + 1 min preinjection steps; injection volumes, 1–2 µL (based on the matrix) in ESI(+) and 5 µL in ESI(−) [4].

The ion source parameters were as follows: sheath gas pressure, 60 arbitrary units (a.u.); aux gas flow, 25 a.u.; sweep gas flow, 4 a.u.; capillary temperature, 300 °C; aux gas heater temperature, 475 °C; spray voltage: 3.5 kV for ESI(+), −3.0 kV for ESI(−). The MS settings were: MS1 mass range, *m/z* 60–900; MS1 resolving power, 17,500 FWHM; AGC target, 1e6; maximum IT, 50 ms; spectrum data type, centroid; the number of data-dependent scans per cycle, 2; MS/MS resolving power, 17,500 FWHM; AGC target, 1e5; maximum IT, 50 ms; spectrum data type, centroid; isolation window, 1 *m/z*; minimum AGC target, 5e2; dynamic exclusion, 2 s; exclude isotopes, on; normalized collision energies: 20, 30, and 40% [4].

Complex lipids. For the separation of complex lipids based on the RPLC mechanism, the following conditions were used: an ACQUITY Premier BEH C18 column (50 mm length × 2.1 mm i.d.; 1.7 µm particle size) with a VanGuard

FIT cartridge (5 mm length × 2.1 mm i.d.; 1.7 µm particle size) (Waters, catalog no. 186009455); column compartment temperature, 65 °C; column flow rate, 0.8 mL/min. For ESI(+), the conditions were as follows: mobile phase A, 60:40 acetonitrile/water with 10 mM ammonium formate and 0.1% formic acid; mobile phase B, 90:10:0.1 isopropanol/acetonitrile/water with 10 mM ammonium formate and 0.1% formic acid; gradient run, 0 min 15% B, 0–0.5 min from 15% to 30% B, 0.5–0.6 min from 30% to 50% B, 0.6–2.8 min from 50% to 80% B, 2.8–3.2 min from 80% to 99% B, 3.2–3.4 min 99% B, 3.4–3.5 min from 99% to 15% B, 3.5–3.7 min 15% B + 1 min preinjection steps. For ESI(−), the conditions were as follows: mobile phase A, 60:40 acetonitrile/water with 10 mM ammonium acetate and 0.1% acetic acid; mobile phase B, 90:10:0.1 isopropanol/acetonitrile/water with 10 mM ammonium acetate and 0.1% acetic acid; gradient run, 0 min 15% B, 0–0.5 min from 15% to 30% B, 0.5–0.6 min from 30% to 50% B, 0.6–2.4 min from 50% to 75% B, 2.4–2.5 min from 75% to 99% B, 2.5–2.9 min 99% B, 2.9–3.0 min from 99% to 15% B, 3.0–3.2 min 15% B + 1 min preinjection steps; injection volumes, 0.3–5 µL (based on the matrix) in ESI(+) and 5 µL in ESI(−) [4].

The ion source parameters were as follows: sheath gas pressure, 60 a.u.; aux gas flow, 25 a.u.; sweep gas flow, 4 a.u.; capillary temperature, 300 °C; aux gas heater temperature, 475 °C; spray voltage: 3.5 kV for ESI(+), −3.0 kV for ESI(−). The MS settings were: MS1 mass range, *m/z* 200–1700 for ESI(+), *m/z* 199–1700 for ESI(−); MS1 resolving power, 35,000 FWHM; AGC target, 1e6; maximum IT, 100 ms; spectrum data type, centroid; the number of data-dependent scans per cycle, 2; MS/MS resolving power, 17,500 FWHM; AGC target, 1e5; maximum IT, 50 ms; spectrum data type, centroid; isolation window, 1 *m/z*; minimum AGC target, 5e2; dynamic exclusion, 2 s; exclude isotopes, on; normalized collision energies: 20% for ESI(+) and 10, 20, and 30% for ESI(−) [4].

Enhancing the acquisition of MS/MS spectra. During LC–MS analysis, we implemented critical rules to enhance metabolome coverage by increasing the acquired MS/MS spectra for metabolite annotation. These rules involved optimizing the number of MS/MS scans per cycle, establishing an appropriate threshold for precursor ion selection, using an exclusion list, employing filters for precursor selection (e.g., isotope exclusion function and charge state), and conducting data-dependent acquisition (DDA) on all measured samples for optimal performance [6]. For each LC–MS platform, an initial exclusion list, consisting of *m/z* values across the entire retention time range (i.e., background contamination, impurities from mobile phases), was generated following the injection of the resuspension solvent. Subsequently, MS1 acquisition and DDA-MS/MS were conducted for all pool QC and SQC samples. The ProteoWizard software was employed to generate an MS2 file with specific parameters (peak picking (vendor): level, 2-2; msLevel, 2-2; threshold, absolute 0.0001 most intense; output, MS2). This MS2 file was then processed by an R script (https://secim.ufl.edu/secim-tools/ie-omics) [7]. An updated exclusion list in CSV format was exported, containing both previously excluded *m/z* values (IE-1) and newly excluded *m/z* values (IE-2), primarily associated with high-abundance precursor ions of metabolites. MS/MS spectra were acquired for high-abundance precursor ions in 15 samples (method IE-1) within each group of samples during the run. Simultaneously, MS/MS spectra for low-abundance precursor ions were acquired for the remaining 20 samples within each group (method IE-2). Both methods (IE-1 and IE-2) alternated during the injection of SQC samples. Additional MS/MS spectra were also acquired using SQC samples and splitting the MS1 mass range into 10 sub-mass ranges to select precursor ions.

Quality control. Due to the high number of samples analyzed, we implemented various quality control measures throughout the metabolomics analyses to ensure data reliability (**Fig. S1**), specifically (i) randomization of the actual samples within the sequence based on the matrix and developmental stage, (ii) injection of quality control (QC) pool samples for each matrix at the beginning, the end, and between every 35 actual samples (for particular matrix), (iii) injection of superior QC samples (SQC), a mix of pool QC samples of all matrices, at the beginning and the end of the sequence and between every 35 actual samples throughout the whole sequence regardless the matrix, (iv) analysis of method blanks, (v) serial dilution of SQC sample (0, 1/16, 1/8, 1/4, 1/2, 1), and (vi) checking the peak shape and the intensity of spiked internal standards and the internal standards added prior to injection [4].

LC–MS data processing

All the LC–MS raw files were converted into ABF format using an ABF converter (https://www.reifycs.com/abfconverter). MS-DIAL v. 4.92 software was used to process converted LC–MS files, including peak detection, deconvolution, alignment, and metabolite annotation (https://systemsomicslab.github.io/compms/msdial/main.html) [8]. The following parameters were used: (i) data collection: MS1 tolerance, 0.01; MS2 tolerance, 0.025; (ii) peak detection: minimum peak height, 20,000; mass

slice width, 0.05; smoothing method, Linear Weighted Moving Average; smoothing level, 2; (iii) MS/MS identification setting: accurate mass tolerance (MS1), 0.01; accurate mass tolerance (MS2), 0.025; identification score cut off, 80%; (iv) alignment: retention time tolerance, 0.05 min; MS1 tolerance, 0.01 Da; peak count filter, 5%; gap filling by compulsion, true.

Metabolites were annotated based on retention time–accurate mass (MS1, MS/MS) match from an in-house spectral library (MSI Level 1) along with MS/MS libraries from various sources (NIST20, MassBank.us, and MS-DIAL MS/MS library v. 15) (MSI Level 2). Complex lipids were annotated using *in-silico* MS/MS spectra available in MS-DIAL software (MSI Levels 2–3). The exported sets were further filtered based on a max sample peak height/blank peak height average <10, an R^2 <0.8 from a dilution series of SQC samples, and a relative standard deviation (RSD) >30% from SQC samples [4]. Data were then normalized using locally estimated scatterplot smoothing (LOESS) with SQC samples injected between 35 actual study samples, and for tissues and feces, based on the amount taken for the analysis. Before statistical analysis, metabolites with more than 50% missing values for each developmental stage and matrix were excluded, and missing data were imputed by replacing 1/5 of the minimal positive values of their corresponding variables for each developmental stage and matrix.

MS-FINDER software v. 3.60 (https://systemsomicslab.github.io/compms/msfinder/main.html) [9] was used for the structure elucidation of N^1-acetylspermidine with the following parameters: mass tolerance (MS1), 0.005 Da; mass tolerance (MS2), 0.005; relative abundance cut-off, 0.1%; LEWIS and SENIOR check, checked; isotopic ratio tolerance, 20%; element ratio check, common range (99.7%); element selection, O, N, P, S; tree depth, 2; local databases + MiNEs + PubChem, checked [2].

ProteoWizard software v. 3.0.22342 (https://proteowizard.sourceforge.io) [10] was used to convert raw LC–MS instrumental files to mzXML format for all platforms.

References

1. Cajka T, Hricko J, Rudl Kulhava L, Paucova M, Novakova M and Kuda O (2023) Optimization of mobile phase modifiers for fast LC-MS-based untargeted metabolomics and lipidomics. Int J Mol Sci 24:1987. https://doi.org/10.3390/ijms24031987
2. Cajka T, Hricko J, Rakusanova S, Brejchova K, Novakova M, Rudl Kulhava L, et al. (2024) Hydrophilic interaction liquid chromatography–hydrogen/deuterium exchange–mass spectrometry (HILIC-HDX-MS) for untargeted metabolomics. Int J Mol Sci 25:2899. https://doi.org/10.3390/ijms25052899
3. Lopes M, Brejchova K, Riecan M, Novakova M, Rossmeisl M, Cajka T, et al. (2021) Metabolomics atlas of oral 13C-glucose tolerance test in mice. Cell Rep 37:109833. https://doi.org/10.1016/J.Celrep.2021.109833
4. Hricko J, Kulhava LR, Paucova M, Novakova M, Kuda O, Fiehn O, et al. (2023) Short-term stability of serum and liver extracts for untargeted metabolomics and lipidomics. Antioxidants 12:986. https://doi.org/10.3390/Antiox12050986
5. Janovska P, Melenovsky V, Svobodova M, Havlenova T, Kratochvilova H, Haluzik M, et al. (2020) Dysregulation of epicardial adipose tissue in cachexia due to heart failure: the role of natriuretic peptides and cardiolipin. J Cachexia Sarcopenia Muscle 11:1614-1627. https://doi.org/10.1002/jcsm.12631
6. Defossez E, Bourquin J, Reuss S, Rasmann S and Glauser G (2021) Eight key rules for successful data-dependent acquisition in mass spectrometry-based metabolomics. Mass Spectrom Rev 42:131-143. https://doi.org/10.1002/mas.21715
7. Koelmel JP, Kroeger NM, Gill EL, Ulmer CZ, Bowden JA, Patterson RE, et al. (2017) Expanding lipidome coverage using LC-MS/MS data-dependent acquisition with automated exclusion list generation. J Am Soc Mass Spectr 28:908-917. https://doi.org/10.1007/s13361-017-1608-0
8. Tsugawa H, Ikeda K, Takahashi M, Satoh A, Mori Y, Uchino H, et al. (2020) A lipidome atlas in MS-DIAL 4. Nat Biotechnol 38:1159-1163. https://doi.org/10.1038/s41587-020-0531-2
9. Tsugawa H, Kind T, Nakabayashi R, Yukihira D, Tanaka W, Cajka T, et al. (2016) Hydrogen rearrangement rules: Computational MS/MS fragmentation and structure elucidation using MS-FINDER software. Anal Chem 88:7946-7958. https://doi.org/10.1021/acs.analchem.6b00770
10. Chambers MC, Maclean B, Burke R, Amodei D, Ruderman DL, Neumann S, et al. (2012) A cross-platform toolkit for mass spectrometry and proteomics. Nat Biotechnol 30:918-920. https://doi.org/10.1038/nbt.2377

Injection order →

Solvent prerun	S	O	L	V	E	N	T	R	U	N
SST, preinjection SQC	SST	SQC								
SQC & dilution series of SQC	SQC	SD	SD	SD	SD	SD	SD			
SQC	SQC									
QC, matrix 1	QC									
Randomized samples, matrix 1, devel. stage 1	S	S	S	S	S	S	S	S	S	S
	S	S	S	S	S	S	S	S	S	S
	S	S	S	S	S	S	S	S	S	S
	S	S	S	S	S					
SQC	SQC									
QC, matrix 1	QC									
Randomized samples, matrix 1, devel. stage 2	S	S	S	S	S	S	S	S	S	S
	S	S	S	S	S	S	S	S	S	S
	S	S	S	S	S	S	S	S	S	S
	S	S	S	S	S					
SQC	SQC									
QC, matrix 1	QC									
Randomized samples, matrix 1, devel. stage 3	S	S	S	S	S	S	S	S	S	S
	S	S	S	S	S	S	S	S	S	S
	S	S	S	S	S	S	S	S	S	S
	S	S	S	S	S					
QC, matrix 1	QC									
SQC	SQC									
…	…									
SQC	SQC									
QC, matrix 10	QC									
Randomized samples, matrix 10, devel. stage 1	S	S	S	S	S	S	S	S	S	S
	S	S	S	S	S	S	S	S	S	S
	S	S	S	S	S	S	S	S	S	S
	S	S	S	S	S					
SQC	SQC									
QC, matrix 10	QC									
Randomized samples, matrix 10, devel. stage 2	S	S	S	S	S	S	S	S	S	S
	S	S	S	S	S	S	S	S	S	S
	S	S	S	S	S	S	S	S	S	S
	S	S	S	S	S					
QC, matrix 10	QC									
SQC	SQC									
…	…									
SQC	SQC									
Blanks	BL	BL	BL	BL	BL	BL	BL	BL	BL	BL
…	…									
SQC & dilution series of SQC	SQC	SD	SD	SD	SD	SD	SD			
SQC, MS/MS acquisition for MS1 sub-mass ranges	SQC	SQC	SQC	SQC	SQC	SQC	SQC	SQC	SQC	SQC

Fig. S1 Schematic overview of the LC–MS data acquisition sequence. The sequence begins with solvent injections to equilibrate the LC–MS platform, followed by a system suitability test (SST) using a mixture of standards, injections of superior quality control (SQC) samples, and a dilution series of the SQC. Each randomized sample set, organized by matrix and developmental stage, is bracketed by injections of matrix-specific QC samples and the SQC (a pooled sample of all matrix-based QCs). The sequence also includes method blank injections to monitor background contamination.

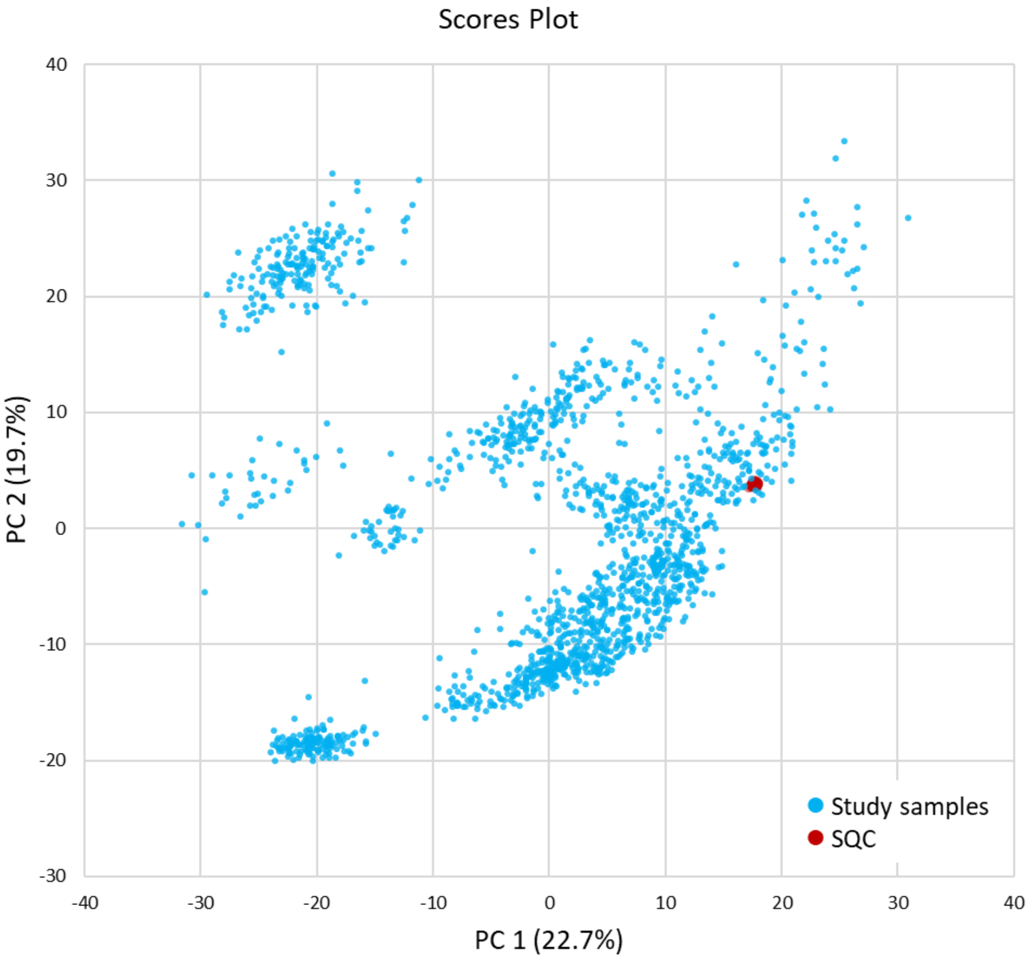

Fig. S2 PCA of 1610 study samples (blue) and corresponding SQC samples (red), based on 851 metabolites. The tight clustering of SQC samples indicates low technical variability and high reliability of the metabolomic analyses.

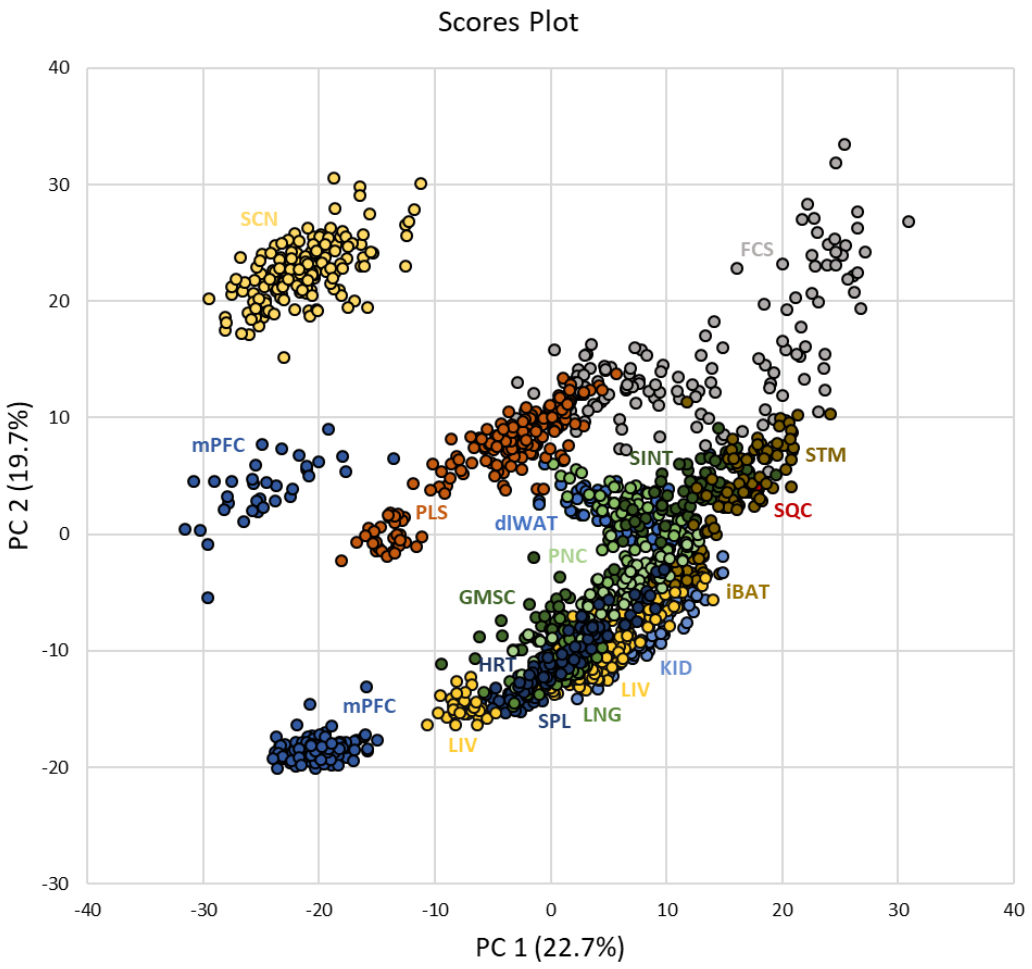

Fig. S3 PCA of study samples colored by a particular matrix and SQC samples based on 851 metabolites. Legend: dorso-lumbar white adipose tissue (dlWAT), feces (FCS), gastrocnemius skeletal muscle (GMSC), heart (HRT), intrascapular brown adipose tissue (iBAT), intrascapular white adipose tissue (isWAT), kidney (KID), liver (LIV), lungs (LNG), medial prefrontal cortex (mPFC), pancreas (PNC), plasma (PLS), small intestine (jejunum) (SINT), spleen (SPL), stomach (STM), suprachiasmatic nucleus (SCN).

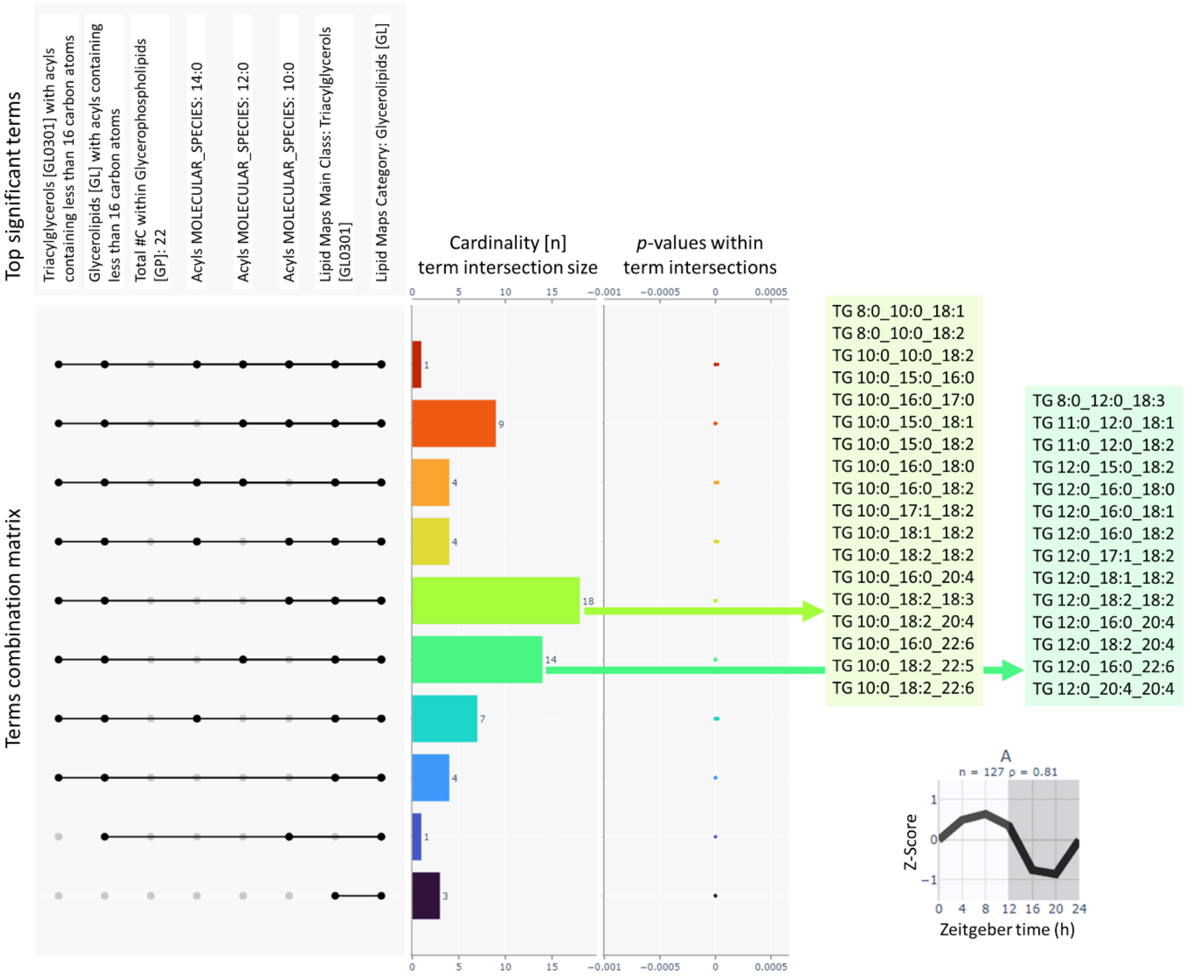

Fig. S4 Example of results from lipid over-representation analysis for plasma at P28, specifically focusing on cluster A. The UpSet plot displays the intersection of over-expressed terms, representing structural characteristics. Cardinality is then sorted based on the total number of term intersections, with each cluster containing lipids that share a particular structural feature. For enrichment analysis, the Fisher exact test (greater) followed by multiple hypothesis testing (FDR (Benjamini–Hochberg) and an alpha level of 0.005 was used.

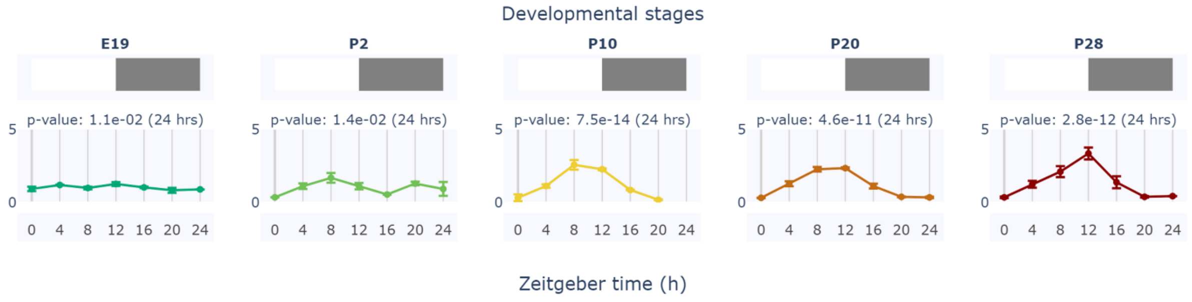

Fig. S5 Expression of clock gene *Per2* in the SCN at different developmental stages.

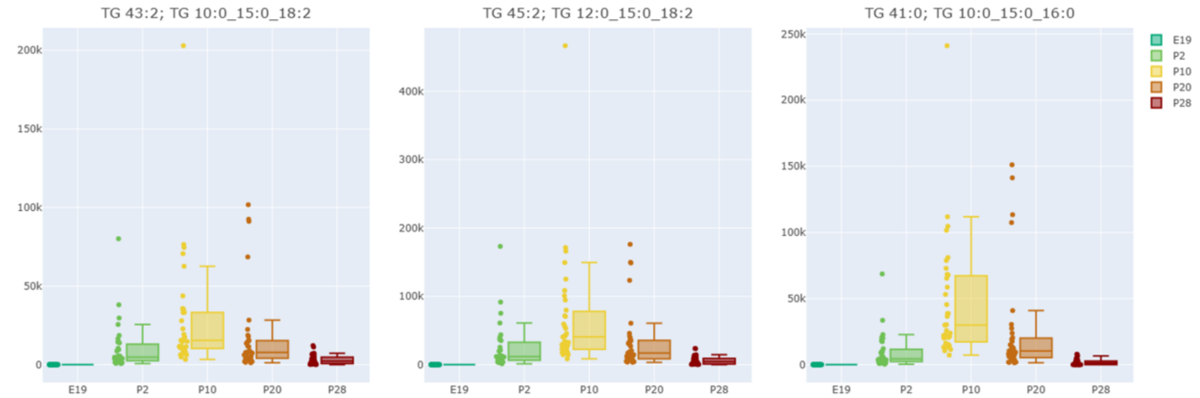

Fig. S6 Profiles of selected plasma triacylglycerols (TGs) containing short-chain saturated fatty acids. TGs were selected based on PLS-DA with a VIP score>1.

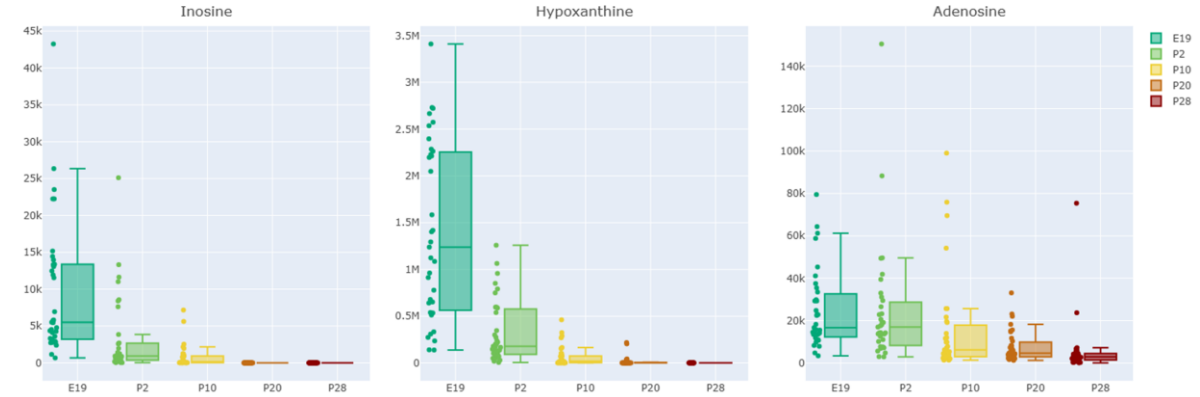

Fig. S7 Profiles of inosine, hypoxanthine, and adenosine detected in plasma based on developmental stage. Metabolites were selected based on PLS-DA with a VIP score>1.

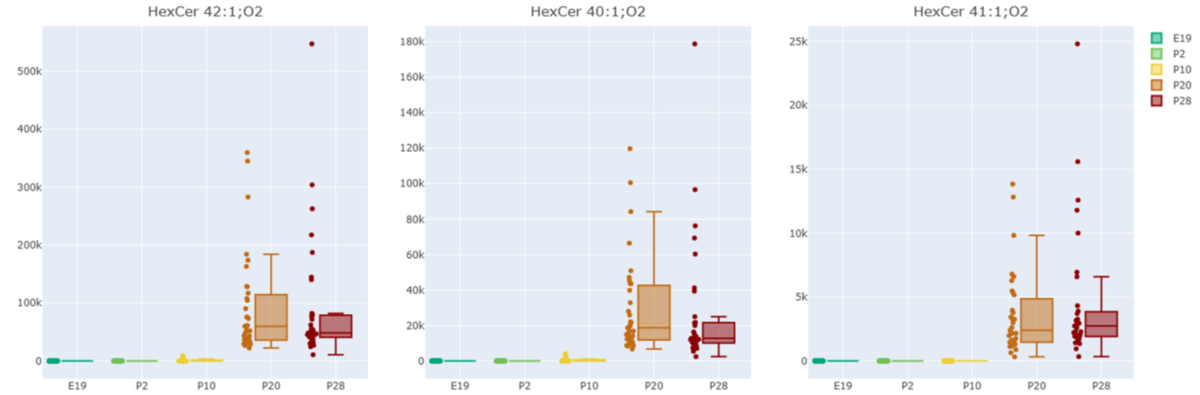

Fig. S8 Profiles of hexosylceramides in mPFC based on developmental stage. Metabolites were selected based on PLS-DA with a VIP score>1.
